# Revisiting the Pathogenesis of X-Linked Adrenoleukodystrophy

**DOI:** 10.3390/genes16050590

**Published:** 2025-05-17

**Authors:** Pierre Bougnères, Catherine Le Stunff

**Affiliations:** 1 MIRCen Institute, Commissariat à l’Energie Atomique, Laboratoire des Maladies Neurodégénératives, 92260 Fontenay-aux-Roses, France; 2 NEURATRIS, 92260 Fontenay-aux-Roses, France; 3 Therapy Design Consulting, 94300 Vincennes, France; 4 UMR1195 Inserm, University Paris Saclay, 94270 Le Kremlin-Bicêtre, France

**Keywords:** X-adrenoleukodystrophy, VLCFA, peroxisomes, oligodendrocytes, neuroinflammation, cerebral demyelination, spinal cord axonopathy

## Abstract

Background: X-ALD is a white matter (WM) disease caused by mutations in the ABCD1 gene encoding the transporter of very-long-chain fatty acids (VLCFAs) into peroxisomes. Strikingly, the same ABCD1 mutation causes either devastating brain inflammatory demyelination during childhood or, more often, progressive spinal cord axonopathy starting in middle-aged adults. The accumulation of undegraded VLCFA in glial cell membranes and myelin has long been thought to be the central mechanism of X-ALD. Methods: This review discusses studies in mouse and drosophila models that have modified our views of X-ALD pathogenesis. Results: In the *Abcd1* knockout (KO) mouse that mimics the spinal cord disease, the late manifestations of axonopathy are rapidly reversed by *ABCD1* gene transfer into spinal cord oligodendrocytes (OLs). In a peroxin-5 KO mouse model, the selective impairment of peroxisomal biogenesis in OLs achieves an almost perfect phenocopy of cerebral ALD. A drosophila knockout model revealed that VLCFA accumulation in glial myelinating cells causes the production of a toxic lipid able to poison axons and activate inflammatory cells. Other mouse models showed the critical role of OLs in providing energy substrates to axons. In addition, studies on microglial changing substates have improved our understanding of neuroinflammation. Conclusions: Animal models supporting a primary role of OLs and axonal pathology and a secondary role of microglia allow us to revisit of X-ALD mechanisms. Beyond *ABCD1* mutations, pathogenesis depends on unidentified contributors, such as genetic background, cell-specific epigenomics, potential environmental triggers, and stochasticity of crosstalk between multiple cell types among billions of glial cells and neurons.

## 1. Introduction

X-linked adrenoleukodystrophy (X-ALD) is a severe disease of the white matter of the nervous system. It is one of the very few neurodegenerative diseases [1] screened at birth in a growing number of US states and other countries [2,3,4,5,6,7,8,9,10,11,12,13,14], which raises hopes for its future prevention. Based on neonatal screening in the USA since 2013, the annual incidence was found to be ~1/10,000 [2,7], much higher than previously thought [15]. Now that X-ALD can be predicted long before its first manifestations appear, a better understanding of its pathophysiology is crucial for targeting therapies to the right cells at the right time in order to prevent, stabilize, or reverse deleterious disease mechanisms.

While developmental myelination seems to proceed normally until 3 years of age, subacute demyelination attacks the brain of a subset of X-ALD patients, most often during childhood (cerebral ALD, cALD). Other patients escape cALD death but develop progressive and severe spinal cord axonopathy (adrenomyeloneuropathy, AMN), most often in middle age. In the late 1970s, the study of brain and spinal cord specimens of patients who had reached terminal cALD or AMN stages provided a thorough description of the white matter lesions characterizing the different X-ALD phenotypes [16,17,18,19,20,21]. In 1976, the discovery of the accumulation of very-long-chain fatty acids (VLCFAs) in brain white matter [22] opened up a biochemical avenue to X-ALD pathophysiology. However, despite the identification of the ATP binding cassette subfamily B member-1 gene (*ABCD1*) as the causal gene in 1993 [23] and the discovery that impaired VLCFA peroxisomal transport is the primary biochemical defect, the mechanisms of X-ALD pathology remained largely obscure. Based on VLCFA accumulation, early authors postulated that X-ALD is a lipid storage disorder, while others tried to establish the toxicity of VLCFA on neurons and glia. In 1990, a single patient showed that allogeneic transplantation of hematopoietic stem cells (HSCs) derived from bone marrow could induce remission of childhood cALD [24], opening the way for dozens of patients to receive the only treatment available for this disease [25] and disrupting our understanding of its mechanisms.

A mouse KO model of the *Abcd1* gene, created in 1997, closely mimics AMN but not cALD [26,27,28,29]. More recently, several new mouse and drosophila models have profoundly influenced our understanding of cALD and AMN pathogenesis. Between 2015 and 2025, gene therapy studies using adeno-associated vectors (AAVs) in the *Abcd1* KO mouse also provided critical information. Recently, X-ALD researchers have started applying technologies, such as induced pluripotent stem cells (iPSCs), to try to characterize the genotypes predisposing patients to cALD or AMN. However, the specific mechanisms leading patients to those two entities mostly remain unelucidated.

In this review, we attempt to use the data available on the different cell populations to discern possible mechanisms underlying X-ALD. This review will raise more hypotheses than certainties. The primary causes and secondary mechanisms of the disease are intermingled over long years in different regions of the central nervous system (CNS), and it is currently impossible to discern which spatiotemporal trajectory of events leads to cALD or AMN. Notwithstanding, at a time when gene therapy attempts are growing in X-ALD, a better knowledge of pathogenesis should provide major support to medical progress.

## 2. Clinical Manifestations

X-ALD was said to give “a constellation of clinical presentations” [30,31], ranging from subacute brain demyelination in young children to a slowly progressive spinal cord axonopathy in middle-aged adults. This review starts with an overview of the clinical pictures because their description sets the agenda for the timing and natural history of CNS lesions.

### 2.1. cALD

Thirty to fifty percent of X-ALD patients in the USA, Europe, or Japan were said to develop the devastating cALD form [3,31,32,33,34]. However, in light of the recent estimation of overall X-ALD incidence [2], it is likely that cALD is less frequent.

Most often, insidious signs appear in children older than 3 years, first as inattention, regression of verbal comprehension and reasoning, or decline in school or behavioral symptoms [35,36]. Within a few months, the child develops apraxia, asomatognosia, disconjugate gaze, unsteady gait, decreased visual perception, poor coordination of movements, defective word hearing and comprehension, seizures, and possibly psychiatric symptoms. Plasma VLCFA measurement enables X-ALD diagnosis [37,38]. The rapid progression of symptoms soon reflects mounting demyelination and inflammation. Thereafter, in almost all cases, the clinical course accelerates further. Deficits become major within a few weeks, leading 90% of children or adolescents to become bedridden, blind, confused, and unable to communicate verbally before entering a vegetative state that leads to death within 3–4 years.

Around 5% of cALD cases occur in adolescents or adults, with symptoms resembling those listed above but whose evolution is slower. The cognitive decline [39] and psychiatric manifestations are often misdiagnosed [40,41,42]. Anecdotical cases of head trauma precipitating cALD have been reported with inflammatory demyelination beginning at the site of the contusion [43,44,45,46].

Intriguingly, for still unknown reasons, 10% of children, adolescents, or men with cALD escape demyelination and neuroinflammation worsening. Nevertheless, after 10–15 years of stability, these patients suffer neurological deterioration of a sudden onset in due to recurring neuroinflammation [47].

### 2.2. AMN

An X-linked adult onset myelopathy associated with adrenal insufficiency was reported in 1976 [20]. A year later, five more cases were described, and the term AMN was coined [16,48]. Increased plasma VLCFA concentrations showed that AMN is an adult form of X-ALD. Virtually all patients with *ABCD1* mutations who reach adulthood develop AMN, usually in their 3rd or 4th decade. Loss of sensation in the legs is followed by a spastic gait with positive Babinski reflex, which progresses inexorably, associated with ataxia, leg pain, impotence, and voiding abnormalities [49,50,51,52]. Within 10–15 years, motor disability of the legs becomes severe, whereas arm and hand deficits remain mild [53,54]. Around 20% of AMN patients develop brain demyelination (cerebral AMN/cAMN) [55]. Those brain lesions either stabilize spontaneously, engendering mild-to-moderate cognitive deficits, psychomotor symptoms, and/or loss of visual memory [39,49], or they enter a phase of worsening symptoms in addition to the preexisting myelopathy [31]. Around 25% of patients with cAMN finally develop a confluent demyelinating brain lesion and catastrophic deterioration [30,56]. Peripheral neuropathy is electrophysiologically detectable in a majority of patients [57], but its clinical contribution is hidden by the prominent myelopathy symptoms.

An 11-year-old male chimpanzee carrying a known *ABCD1* mutation developed a phenocopy of cAMN including leg weakness, drooling, occasional erratic behavior, inability to focus, high plasma VLCFAs, and typical magnetic resonance imaging (MRI) features. As symptoms progressed, he had difficulty swallowing, dragged his legs when moving, and had to be euthanized [58]. Unfortunately, this primate did not have descendants that would have allowed for the study of disease mechanisms as a proxy for humans.

Although ALD is an X-linked disease, slowly progressive AMN symptoms occur in ~65% of heterozygous women by the age of 60 years [31,47,59,60] due to the inactivation of the non-mutated X chromosome and the mosaic expression of X-linked genes in neural cells [60,61,62,63]. These women rarely experience cerebral involvement and adrenal insufficiency [47,64].

### 2.3. Adrenal Insufficiency

At diagnosis, 80% of boys or adolescents with cALD and 70% of AMN patients have hormonal evidence of clinically silent adrenal insufficiency [65,66,67,68,69].

## 3. X-ALD Patients’ CNS Lesions in Postmortem Studies and MR Images

A few brain and spinal cord specimens, studied 40–70 years ago, provided unique information about CNS lesions. Surprisingly, despite the existence of active neurobiobanks in Europe and the USA [70], post mortem analyses have no longer been performed in X-ALD patients since the end of the 1970s, in contrast with other CNS diseases [71], except in a handful of cases. This is probably due to the very small number of X-ALD patients who die every year. Many unsolved questions, however, would benefit from cell studies of the brain and spinal cord using immunofluorescence for cell markers, electron microscopy and transcriptomics.

### 3.1. Brain Lesions in Patients with cALD or AMN

Brain lesions were initially described in 18 patients with cALD who died between 1955 and 1974 [17,19]. Macroscopy revealed a variety of lesions in the corpus callosum, fornix, hippocampal commissure, posterior cingulum, the tracts passing through the posterior limb of the internal capsules, lateral cerebral peduncles, pons, pyramids, corticospinal tracts, and/or optic nerves and tracts. A confluent lesion usually extended into the opposite hemisphere via the splenium of the corpus callosum. Severe and symmetrical white matter lesions had developed in the parietal, occipital, and posterior temporal lobes, as well as asymmetrical lesions in the frontal cortex, associated with loss of subcortical white matter and variable ventricular dilatation. Often, the cerebellum and sometimes cerebellar peduncles were also involved. In one case, white matter destruction was almost complete after only 9 months of clinical evolution. No obvious macroscopic lesions were described in the spinal cord of young patients with cALD who died. Histopathology of five brains showed marked disparity across regions [72] with contiguous white matter areas [73]: (i) intact zones showing densely packed myelinated axons and normal-looking oligodendrocytes (OLs); (ii) zones of limited demyelination showing occasional astrocytes and lipid-containing macrophages; (iii) zones of active demyelination with loss of axons and myelin, variable loss of OL, increased proportions of naked axons, varying numbers of reactive astrocytes, numerous microglia, lipid-laden macrophages containing myelin debris, perivascular cuffs of lymphocytes associated with interfascicular OLs, and “extraordinarily severe” perivascular or interstitial infiltration of foamy macrophages; (iv) “chronic active demyelination” and completely demyelinated zones of white matter populated by few astrocytes; (v) chronic gliotic lesions with no axons, no OLs, sparse lymphocytes and perivascular macrophages, and variable astrogliosis. The very prominent and distinctive feature of cALD is the often-intense lymphocytic infiltrates in the lesions, which are not typically seen in other metabolic leukodystrophies.

Electron microscopy reveals characteristic cytoplasmic inclusions (“spicules”) in OLs, perivascular macrophages, and/or microglia but not in astrocytes. Spicules are straight and curved striated structures made of degenerated myelin [17,20,22,74,75,76,77,78,79,80], which are also observed in 15% of Schwann cells surrounding myelinated axons in the adrenals [19,81].

AMN patients show a variety of mild brain lesions. A few of them had no white matter lesions [49], but almost all showed microscopic dysmyelinated foci, activated microglia and macrophages, and relative to total sparing of axons and OLs [49]. Reactive astrocytes and lymphocytes were absent or minimal. Other patients had confluent myelin losses in the cerebrum and to a lesser extent the cerebellum, but no inflammation [49]. Still others develop cAMN, with inflammatory demyelinating lesions and axonal loss, resembling cALD but much more localized [49]. Mixtures of lesions coexisted [49]. Corticospinal tract degeneration was observed in the midbrain, pons, medulla, and spinal cord, with mild reactive astrocytosis and a few lymphocytes [49]. Noninflammatory, bilateral loss of axons and myelin affects the medial and lateral lemnisci, brachium conjunctivum, middle and inferior cerebellar peduncles, optic system, and geniculo-calcarine tracts [16,30].

### 3.2. Spinal Cord Lesions

In its pure form, AMN is primarily and almost exclusively a spinal cord disease. The only data we have come from ten adult male patients who were autopsied ten to twenty years after diagnosis at terminal stages of AMN disease [16,18,20,21,30,49,82,83], who showed spinal cord atrophy. To our knowledge, earlier stages of myelopathy have not been studied in AMN patients who died from incidental causes.

The most prominent and consistent lesions in patients who died from AMN were bilateral, symmetrical degeneration of the long ascending and descending tracts of the spinal cord. The cervical fasciculus gracilis, the dorsal spinocerebellar tract, and the lumbar lateral corticospinal tract are the three structures most severely affected by axon degeneration and myelin loss [49]. The ascending fasciculus gracilis originates from first-order neurons located in dorsal root ganglia (DRG). The heavily myelinated axons of the fasciculus gracilis convey conscious proprioceptive, tactile, and vibratory ipsilateral information from the lower trunk and extremities. These axons synapse in the medulla oblongata gracilis nucleus to second-order neurons, from which axons (medial lemniscus) decussate, passing through the pons and midbrain to terminate in thalamus. Third-order thalamic neurons travel through the posterior limb of the internal capsule to reach the sensory cortex (Brodmann areas 3,1,2). In AMN patients, the fasciculus gracilis shows a prominent loss of myelinated fibers and OLs [18], associated with axonal atrophy and very thin or disintegrating myelin sheaths [49,82]. Axonal loss is commensurate to myelin loss. The other prominently affected structure in AMN is the dorsal (posterior) spinocerebellar tract, located on the lateral surface of the cord. The axons of this tract originate from the DRG axons and convey unconscious proprioceptive information through the inferior cerebellar peduncle to the cerebellar cortex. The third spinal cord tract prominently affected in AMN is the descending lateral corticospinal (pyramidal) tract at the lumbar level. These axons originate from first-order neurons located in the cerebral motor cortex. After decussation, they constitute the pyramidal tracts, which travel downwards over the entire length of the spinal cord white matter to finally synapse on second-order motor neurons in the ventral horns of lumbar spinal cord. Through this connection, they control voluntary discrete skilled movements of the distal parts of the limbs.

It is remarkable that the axonal degeneration of the three structures most affected in AMN myelopathy starts very far from the first-order neurons of origin located either in DRG (ascending tracts) or the cerebral cortex (descending tracts). Because the cell bodies of these parental neurons are intact and in normal numbers, AMN is considered a “dying back” axonopathy, with the longest vulnerable axons in the spinal cord suffering the greatest damage.

Microglia are present in lesions, but there are no infiltrates of circulating inflammatory cells, indicating that myelopathy is a non-inflammatory process. Cuffs of perivascular macrophages are common in the degenerating tracts [49,82].

We cited above the three structures that show consistent atrophy at a terminal stage of the disease and are consistent with the clinical manifestations of AMN. This does not exclude the existence of less severe axonal abnormalities in other tracts of the spinal cord and possibly of the brain.

No obvious macroscopic lesions were described in the spinal cord of young patients who died from cALD.

### 3.3. Peripheral Neuropathy

The few studies on peripheral nerves in AMN [16,18,20,82,84,85,86] highlight variable and mild lesions of chronic axonal atrophy with secondary demyelination [49]. Spinal nerve roots and ganglia, sciatic, popliteal, and ulnar nerves are unremarkable, even when myelopathy is severe. Sural and peroneal nerves show loss of large and small diameter myelinated fibers, endoneurial fibrosis, and thin myelin sheaths. Inclusions in Schwann cells or endoneurial macrophages are seen in peroneal nerves. Sural nerves suffer more specific involvement of large myelinated fibers [87], causing deficits in position and vibratory sensations. Peripheral nerves harbor no inflammatory cells. Plexi, notably the lumbar plexus, lose myelinated fibers, with myelin ovoids and some endoneurial fibrosis [49]. The anterior roots are normal or contain a few perivascular and endoneurial lymphocytes without macrophages [49]. Clusters of thinly remyelinated axons in the teased fiber analysis are consistent with primary axonal atrophy [88]. In DRG, onion bulb formations are observed without loss of neurons [49,89].

## 4. Imaging of CNS Lesions

During the follow-up of a patient’s brother with elevated VLCFAs or a child found positive at neonatal screening, MRI is able to detect early demyelinating lesions generally localized in the splenium of the corpus callosum (a region with a very high density of OLs), which progress to involve the adjacent parieto-occipital white matter [14,31,90,91,92]. At an early stage, brain MRI shows more intense T2- and fluid-attenuated inversion recovery (FLAIR)-sequence signals. At more advanced stages, typical confluent and symmetrical areas of T2 hyperintensity reflect demyelination in the splenium of the corpus callosum, parieto-occipital or frontal white matter, or pyramidal tracts within the internal capsules, pons, and brainstem. Those lesions initially had no or minimal enhancement on T1 sequences after gadolinium administration [31,93,94]. Gadolinium extravasation, a hallmark of neuroinflammation and blood–brain barrier (BBB) breach, can occur early when demyelinating lesions are still restricted to the corpus callosum or pyramidal tracts but is often seen later, once the demyelinating lesions have extended [31,93,94]. In the few cases of “arrested cALD”, MRI is stabilized and gadolinium enhancement disappears for a decade or more [95,96].

Magnetization transfer or diffusion tensor-based imaging sequences can detect abnormalities in spinal cord tracts [65,97,98]. Late spinal cord MR images may show some atrophy, but no demyelination or gadolinium enhancement. In patients with “pure” AMN, brain MRI remains normal or shows subtle abnormalities, such as moderately increased signal intensities on FLAIR and T2 sequences in the pyramidal tracts in the internal capsules, brainstem, and/or pons [31]. In some patients, the pyramidal tract signals may become as intense as in cALD [31]. In patients developing cAMN [55], demyelinating lesions often start at the posterior limb and splenium of the corpus callosum [56]. Those brain lesions may enter an active phase of neuroinflammation with gadolinium enhancement [31]. We have previously seen that 25% of patients with cAMN finally develop confluent brain demyelination [30,56].

## 5. The *ABCD1* Gene and Its Expression

### 5.1. The X-Linked ABCD1 Gene

Starting in 1990, positional cloning of the X-ALD gene in the Xq28 region was attempted in vain for a hundred patients, based on the erroneous hypothesis of a contiguous gene syndrome involving the R/GCP color vision gene (personal information). In 1993, the gene responsible for X-ALD (OMIM300100) was identified in an Irish patient, whose complex DNA rearrangement serendipitously positioned the *ABCD1*-coding sequence close to the R/GCP locus. The newly discovered *ABCD1* gene was doubtfully labeled by the journal *Nature* “a putative X-ALD gene” [23] but soon proved to be the true X-ALD-causative gene [99]. Hundreds of mutations were reported. While writing this review, we updated the previous counts [100] and found a total of 172 variants annotated as “likely pathogenic” and 825 as “pathogenic” in the *ABCD1* registry (https://adrenoleukodystrophy.info, accessed on 15 May 2025). The de novo mutation rate ranged from 4 to 19% [32,101]. 

### 5.2. Individual Variability of Disease Phenotypes

More strikingly than in many single-gene diseases, *ABCD1* mutations do not govern the individual variability of clinical manifestations. Beyond the “one gene, one disease” paradigm, large deletions or frameshift mutations leading to the complete absence of the ALD protein (ALDP) can be associated with mild AMN phenotypes as well as with cALD [102]. Devastating childhood cALD and late adulthood AMN may co-exist within the same family [103,104,105,106], whereas the few studied monozygotic twins showed less discordant phenotypes [107,108,109,110]. Therefore, the implication of “modifier genes” was suspected, as in all monogenic diseases with variable penetrance. Although a large segregation analysis suggested the presence of a single “major” autosomal modifier locus [111], attempts to identify a modifier variant among 16 candidate genes, including some involved in inflammatory processes, had no success [15,112,113,114,115,116], as reviewed in [15]. More candidates will come from transcriptomic studies of patients’ CNS specimens [80] or induced pluripotent stem cells (see Section 10.2). Unless they are relatively common and have a major phenotypic effect, modifier-gene variants (likely to exist) may only be identified if agnostic whole-genome analysis studies are able to recruit and compare thousands of well-defined patients with cALD with thousands of patients with “pure” AMN [117,118]. Unraveling putative environmental factors or variable epigenetic blueprints would also require very large cohorts, currently out of reach. Comparing hundreds could only reveal “major” modifier genes, environmental exposures, or epigenomic marks, if they exist.

Among environmental exposures, viral infections, which could interfere with VLCFA metabolism and peroxisomes [119,120], might be suspected to trigger cALD. There is, however, no direct evidence for that, and this remains to be studied. Intriguingly, neuropsychiatric manifestations were not only reported following infection with neurotropic influenza A variants but also after non-neurotropic H1N1 virus infection, especially in children [121]. Influenza infection may enter the brain through several routes [122,123] or send deleterious signals to the brain via sensory neurons [124] and trigger neuroinflammation or associated chronic alterations in the CNS [125]. The coronavirus disease-2019 (COVID-19) pandemic should enable testing of whether cALD cases were increased in patients with moderate-to-severe COVID-19 and might be immune-mediated [126]. Indeed, while COVID-19 is very rarely neuroinvasive [127], its impact on the brain might be immune-mediated [126] and cause glial neuroinflammation [128,129,130]. It would therefore be of interest to study whether or not the relative cALD frequency has increased during or after the pandemic in patients with mutated *ABCD1*.

Beyond identifiable causes, a large part of phenotype variability across patients with the same *ABCD1* genotype may “simply” be due to stochasticity, a factor hitherto considered “noise” by Cartesian researchers. Actually, stochasticity rules all living cells through fluctuations in gene expression, molecular interactions, and metabolic fluxes, even systems governed by deterministic laws, such as monogenic genetics [131,132,133,134]. Indeed a given *ABCD1* mutation is not expected to lead to the same phenotypes in different people, since disease mechanisms involve multiple metabolic and inflammatory processes interacting throughout years in tens of billions of different cells [135]. *Abcd1*^−/−^ mice, despite being congenic littermates living in the same cage, show variable motor deficits at different ages, thereby supporting the importance of stochasticity for determining phenotypes [136].

In summary, an *ABCD1* mutation is sufficient to cause “pure” AMN, while being necessary but not sufficient to cause cALD or cAMN.

### 5.3. ALDP, the ABCD1-Encoded Protein

ALDP is a 745-amino-acid-long ATP-binding cassette (ABC) transporter [137]. Many *ABCD1* missense mutations affect ALDP folding and stability [138,139]. Molecular modeling of mutated ALDP has suggested the mechanism of its dysfunction [140]. ALDP is synthesized on free cytosolic ribosomes then targeted to peroxisomes via a peroxisomal biogenesis factor-19 (Pex19p)-dependent pathway, then inserted post-translationally into the peroxisomal membrane [141]. Its N-terminal part contains a transmembrane domain, and its C-terminal part contains a single ATP-binding domain at the cytoplasmic surface of the peroxisomal membrane [142]. ALDP transports VLCFA across the peroxisomal membrane [143], preferentially as saturated and monounsaturated VLCFAcyl-CoA, notably C22:0–CoA, C24:0–CoA, C26:0–CoA, and C26:1–CoA [143,144,145,146]. Transport involves cooperative binding of two VLCFAcyl-CoAs, ATP binding, and ALDP homodimerization [147], followed by outward-facing ALDP conformational change allowing for the release of VLCFAcylCoA into the peroxisome matrix [148], where VLCFAcyl-CoA undergoes peroxisomal β-oxidation [149]. In humans, two other ATP-synthase ABC transporters can transport VLCFAcyl-CoA into peroxisomes: adrenoleukodystrophy-related protein (ALDR), coded by *ABCD2*, and 70 kDa peroxisomal membrane protein (PMP70), coded by *ABCD3* [146]. Consistently, the residual peroxisomal β-oxidation of VLCFA is ~20% of normal in X-ALD fibroblasts [150,151] and can be restored by *ABCD2* or *ABCD3* overexpression [152,153]. X-ALD pathological manifestations are thought to reflect the absence of mutated ALDP transporter activity, but in some genetic or non-genetic contexts, it could be linked with the dominant negative effect of abnormal ALDP [154]. To this point, it should be noted that studies of mutated *ABCD1* expression have been limited to mRNA quantification [155], and, to our knowledge, mutated ALDP in the CNS of X-ALD patients has never been quantified.

### 5.4. ALDP Expression in Human Cells

P. Aubourg’s team found ALDP to be highly expressed in the brains of four infants who died from non-metabolic pathologies: in astrocytes, microglia, perivascular macrophages, endothelial cells, and in a subset of OLs located in the corpus callosum, internal capsules, and anterior commissure [156], the regions where demyelination is first seen on MR images of cALD. ALDP expression varied in other OL subpopulations, weak in subcortical white matter, cerebellum, brainstem, and pons, and absent in cerebral cortex or cerebellar neurons. In contrast, two peroxisomal enzymes involved in β-oxidation of VLCFA, acyl-CoA oxidase, and catalase were expressed in all human and mouse brain cells, including neurons and endothelial cells [156]. According to another study on four adults who died of non-metabolic diseases at 22–79 years of age [157], ALDP was expressed in astrocytes and microglia in subcortical and cerebellar white matter but almost absent from the internal capsules, corpus callosum, and the corticospinal tract. ALDP expression was weak in OLs, abundant in neurons of the hypothalamus, basal nucleus of Meynert, periaqueductal grey matter, and loci cerulei; moderate in the neurons of the thalami and dorsal nucleus of the vagus; sparse in neurons of the frontal temporal, parietal, and occipital cortices, hippocampus, and amygdala; and absent in neurons of the caudate, subthalamic, dentate and olivary nuclei, the substantia nigra, and the cerebellum. ALDP expression was found in 40% of thoracic DRG neurons. ALDP was also expressed in endothelial cells, ependymocytes of the ventricular system, choroid plexus, Bergmann glia in the cerebellum, and corticotropic cells in the pituitary. The ALDP expression pattern in five fetal brains was comparable to that in adult brains. Perivascular macrophages showed abundant ALDP expression in a patient with viral encephalitis [156]. Those studies did not quantify the proportion of ALDP-expressing cells in those brains, and we have not been able to find reliable information about ALDP expression in normal spinal cord cells.

In blood cells from ten healthy male adults, *ABCD1* mRNA levels were the highest in granulocytes, followed by monocytes, intermediate in B cells, and low in natural killer cells and T cells. Monocytes and granulocytes deprived of functional ALDP and barely expressing *ABCD2* may thus be the most severely affected immune cells in X-ALD patients [158]. The highest ALDP expression was located in the adrenal cortex fasciculata and reticularis zonae.

### 5.5. VLCFA Metabolism and Myelin

VLCFAs not used for myelin synthesis undergo β-oxidation in OL peroxisomes. By binding to ATP, ALDP transports VLCFAcyl-CoA into the peroxisomal matrix [143,159,160], where they are β-oxidized into medium-chain fatty acids (MCFAs) and MCFAcyl-carnitines [161], propionyl-CoA, and acetyl-CoA. VLCFAs cannot enter mitochondria; therefore, mitochondria cannot β-oxidize them. All of these metabolites must be transferred into mitochondria for full β-oxidation into CO_2_ and H_2_O because peroxisomes lack a Krebs’ cycle [162]. Peroxisomal β-oxidation of VLCFAs has a unique degradative function [149]. Abnormal VLCFA (C > 22) accumulation was first documented in the brains of a young adult and five children with cALD [163,164]. The white matter and myelin of actively demyelinating areas showed major VLCFA accumulation in glycerolipids, notably phosphatidylcholine (PC) and cholesterol esters (CEs). White matter areas appearing histologically intact also showed VLCFA accumulation in PC and more limited VLCFA increases in CEs. The authors concluded that abnormal VLCFA metabolism preceded demyelination [163,164] because CE-synthesis enzymes are ten times more active than CE-hydrolysis enzymes [165]. In the intact brain white matter of cALD patients, C26:0 is 1.5-fold higher than in “pure” AMN [155]. In human X-ALD brains, VLCFA accumulation occurs mostly in astrocytes, microglia, endothelial cells, and OLs located in the corpus callosum, internal capsules, and anterior commissure [156]. VLCFA accumulation has also been studied in induced pluripotent stem cells (iPSCs) derived from X-ALD patients (see Section 10.2). In the 2′,3′-cyclic nucleotide 3′-phosphodiesterase (CNP)-Pex5 mouse brain, VLCFA accumulation is prominent in OLs, notable in astrocytes, and non-existent in neurons [166]. Cytoplasmic inclusions (“spicules”) made of cholesterol, phospholipids, and gangliosides esterified with saturated VLCFAs [22] were found in human brain OL and macrophages and in adrenal cells or Schwann cells [167].

Over the years, researchers have proposed hypotheses attempting to link VLCFA accumulation with neuropathology. Myelin physical and chemical properties were first suspected. Indeed, a highly saturated VLCFA content can decrease myelin fluidity, and myelin damage can be triggered by small changes in its constituents [168]. Fatty acyl chain length alters binding constants of several membrane proteins [169], making it tempting to speculate that VLCFAs might alter neural cell membrane structure, stability, and function [170]. Other hypotheses about the deleterious consequences of VLCFA accumulation will be discussed below. The most recent X-ALD expert opinion [171] is that “the accumulation of VLCFA has vexed the field since their discovery 50 years ago” [22]. The challenge remains to establish whether and how VLCFA accumulation interferes with axonal loss, demyelination, or neuroinflammation, the main X-ALD mechanisms. In a recent study, we observed a strong correlation between C26:0-lysophosphatidylcholine (LPC) content in spinal cord and cerebellum with motor performances of *Abcd1*^−/−^ mice, suggesting that the level of VLCFA accumulation is associated, directly or indirectly, with the pathological phenotype [172].

### 5.6. Mutant Abcd1 Animal Models

Several animal models of *Abcd1* deficiency have been created [173,174].

#### 5.6.1. *Abcd1*-Knockout (KO) Mouse

**Figure 1:**
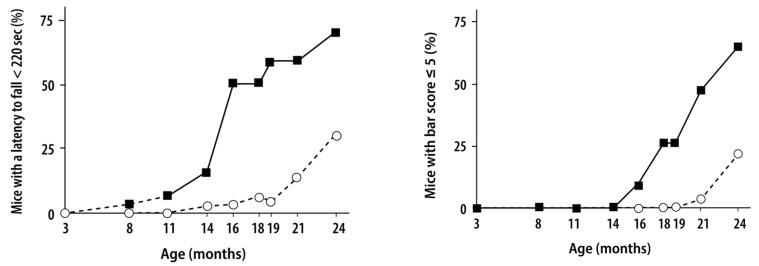
Motor performance of *Abcd1* KO mice (black squares) compared with wild type littermates (open circles) tested with rotarod (**left**) or bar walking score (**right**). The percentages of mice falling more quickly from the rotarod or having a low score on the bar is shown from 8 to 24 months of age [176].

*Abcd1*-knockout mice created in the late 1990s closely mimic the late and progressive spinal cord axonopathy of “pure” AMN. VLCFA accumulation occurs, but not brain lesions [26,27,28,29] or peripheral neuropathy [175]. We recently studied 40 *Abcd1*^−/−^ mice until 24 months of age [172,176]. They were male mice, thus the superscript ^−/−^ can also be read ^−/y^, as sometimes written in the literature Our results confirmed and extended previously reported findings in smaller numbers of mice [26,27,28,29]. Motor performance remained normal in all mice until 16 months of age. Motor and coordination deficits predominantly affecting the hind legs appeared in ~25% of *Abcd1*^−/−^ mice at 18 months, an age comparable to ~50 years in humans [177] (Figure 1). The motor deficit strikes more mice and worsens between 18 and 24 months of age. At 24 months, ~70% of mice had severe motor manifestations but 33% still showed no clinical pathology (Figure 1). This individual variation cannot be explained by genetic or environmental factors in congenic littermates living in the same cage. No brain or spinal cord lesions were visible macroscopically or using light microscopy, and no significant demyelination or neuroinflammation was detected. OL and astrocyte counts were normal in the dorsal columns of the spinal cord, corpus callosum, and cerebellum [172,176]. The only spinal cord abnormality revealed using electron microscopy was axonal degeneration with organelle accumulation and dense body (amyloid precursor protein, (APP)) deposits ([29], personal observation). Myelin sheaths were apparently normal. There were clear signs of microglial activation in the spinal cord based on cell shape; pro-inflammatory markers were not elevated. However, cultured microglia appeared to be primed, as they reacted much more strongly to lipopolysaccharide (LPS) challenge [178]. Surprisingly, upregulation of signaling molecules for phagocytosis (including milk fat globule-epidermal growth factor 8 (MFGE8), triggering receptor expressed on myeloid cells 2 (Trem2)) in spinal cords and in vitro occurring at an early stage, several months before synapse loss and axonal degeneration. Because *Abcd1*^−/−^ mice have not been followed until death, it is not known whether corticospinal and spinocerebellar tract atrophy would be seen, as in the postmortem spinal cord of AMN patients. Experimental results from A. Pujol’s laboratory demonstrated overproduction of redox free radicals altering mitochondrial functions in brain white matter or cultured cells [80,136,179,180,181,182,183,184,185,186,187,188,189]. Those observations, however, do not explain why the brain was unaffected, despite its abnormal redox status. In the cited studies, ultrastructural observations consistently show that axonal damage was the first pathological event followed by myelin degeneration [190]. It is regrettable that peroxisomes were neither counted nor studied in the spinal cord or brain cells of *Abcd1*^−/−^ mice. The potentially major role of OLs in inducing the spinal cord pathology without brain pathology in patients or mice will be further discussed below.

#### 5.6.2. Other ABCD1-Mutant Animals Have Also Been Studied

The *abcd1* mutation in zebrafish demonstrated a major and likely primary role of OLs, with VLCFA accumulation, altered myelination, increased apoptosis, and abnormal OL patterning, cell death, early motor impairment, and shortened survival [191]. Human *ABCD1* expression in OLs reversed the zebrafish disease [191].

Drosophila *abcd1* mutants were found to have brain VLCFA accumulation, locomotor impairment, and other abnormalities [192]. Although flies have no myelin, their glial wrapping somewhat resembles vertebrate myelin [193]. These mutant flies had fewer peroxisomes, demonstrating an *ABCD1* role in peroxisomal biogenesis [194]. Based on this observation, it would be of interest to know whether *ABCD1*-mutated patients or *Abcd1*^−/−^ mice have fewer or abnormal peroxisomes.

Loss of the *pmp-4* gene, the *Caenorhabditis elegans* ortholog of *ABCD1*, induces VLCFA accumulation, axonal degeneration, and locomotor dysfunction [195].

#### 5.6.3. *Abcd2* KO and *Abcd1*: *Abcd2* Double KO Mice

Other interesting models are the *Abcd2* KO and the *Abcd1:Abcd2* double KO mice [190,196]. Electron microscopy in *Abcd2* KO mice showed the first signs of spinal cord axonal pathology at 12 months, yet no histological abnormalities. Histological abnormalities in spinal cord dorsal and ventral columns became evident at 20 months, while atrophy or death of Purkinje cells was detected in the cerebellum. The brain of *Abcd2* KO mice showed no significant difference compared to wild type littermates. At 12 months of age, *Abcd2* KO mice already displayed a marked impairment in their motor performance, which aggravated up to 20 months. *Abcd1: Abcd2* double KO mice had an earlier onset of symptoms with more severe motor deficits (at 20 months of age, 6 out of 11 mice were not able to perform motor tests), axonal degeneration, and myelin alterations, but no cerebral pathology.

## 6. Main Characteristics of Neural Cell Populations in Health and VLCFA-Related Diseases

Many cell populations in the CNS and peripheral nerve system (PNS) are likely to contribute to X-ALD pathology as primary culprits, players, and/or victims aggravating disease mechanisms. From early childhood to late adulthood, the disease evolves more or less rapidly in affected regions of the nervous system and spares others. We have seen that this spatiotemporal evolution of dysfunction and cell loss follows a process that maintains its unknowns and is not directly determined by the *ABCD1* gene defect. Complex disease mechanisms involve a number of cell-specific functions in the context of multiple crosstalks among tens of billions of glial cells and neurons, whose overall organization is, as for all neurodegenerative diseases, not easy to follow. Below, we review the various cell types that can be suspected of playing a role in X-ALD pathogenesis, in the light of recent experimental studies. Beyond pathogenesis, knowing the characteristics of cell types in health and diseases is of utmost importance to target the right cells for efficient gene therapy strategies.

### 6.1. OLs

Mature OLs are abundant alongside axon tracts, each of them enwrapping tens of axons with myelin sheaths [197,198]. OL progenitor cells (OPCs), located in the ventricular zones of the brain and spinal cord, continuously proliferate to renew OLs. OLs have a long lifespan in humans and mice [199,200,201,202], where their populations are heterogeneous [203]. Myelin synthesis requires highly productive biosynthesis machinery to generate and deliver large amounts of newly synthesized lipids. Brain OLs account for the majority of VLCFA synthesis [166,204] by using their ELOVL1 [205,206,207,208]. They also have high cholesterol synthesis capacity, notably in the spinal cord [209]. OL peroxisomes are largely responsible for VLCFA synthesis [204] and degradation [166] in the adult brain.

OLs incorporate various proportions of LCFAs and VLCFAs into glycerolipids, glycerophospholipids (phosphatidylcholine), and sphingolipids (sphingosine, sphingomyelin, ceramide), all of which are myelin constituents. Myelin is composed of 35% cholesterol, 25% phospholipids, and 40% sphingolipids [210,211,212]. Half of myelin sphingolipids contain VLCFAs (C > 22) [213,214,215]. In the mouse brain, C24 sphingomyelin accounts for 25% of sphingomyelin [216]. Because VLCFAs are rare fatty acids (FAs) that make up only 5% of the total body FAs, their rich content of normal myelin is remarkable. VLCFAs increase membrane stiffness, thereby decreasing fluidity, and they are likely critical for proper myelin function [191,213,214,217], myelination [218], myelin maintenance [207,213,215], and neuron polarity during CNS development [219]. Throughout life, myelin is continuously and slowly renewed [220,221]. Myelin undergoes structural changes [222] that are likely driven by, and interact with, motor skill learning [223,224,225], memory [226], social experiences [227,228,229] including early adversity [230,231], ageing [232], and environmental cues [233,234,235]. Myelin is a huge FA reservoir for the CNS. OL macroautophagy, the degradation of myelin lipids in lysosomes that liberates FAs for β-oxidation [236] and synthesizes new myelin lipids [237], maintains myelin sheaths. The dysregulation of macroautophagy leads to neurodegeneration [238]. Since myelin is continuously degraded, OLs β-oxidize myelin LCFAs in their mitochondria to produce energy and recycle non-oxidized LCFA to make new myelin. In parallel, OLs use their peroxisomes to β-oxidize VLCFAs and to detoxify the hydrogen peroxide produced during FA β-oxidation [239]. In addition to peroxisomes, lipid droplets in the brain have only recently gained attention [240,241]. These organelles engage in non-vesicular lipid transport [242,243] by stockpiling FAs in the form of triglycerides and cholesterol esters and transferring VLCFAs to peroxisomes for β-oxidation [244,245,246]. This non-vesicular lipid transport in OLs is an important contributor to myelin growth [247,248,249,250].

OLs are rich in peroxisomes, their content several times that of neurons or astrocytes [166,251,252,253,254]. Peroxisomes are found in OL processes adjacent to the outer cytoplasmic myelin mesaxon [255]. They are abundant in all cytoplasmic regions of myelin sheaths, and OL “myelinic channels”, the cytoplasmic channels of non-compacted myelin [253,254]. Damaged or superfluous peroxisomes are removed mainly by pexophagy, which is the selective autophagy of peroxisomes. It can affect other autophagy pathways for mitochondria (mitophagy) or protein aggregates (aggrephagy), thereby reducing the clearance of many substrates [256] that can lead to cell degeneration [257]. Although our knowledge of the peroxisome role in disease has increased in recent years [149], numerous unknowns regarding pexophagy remain to be addressed [258,259]. High VLCFA concentrations induce peroxisome loss and cell death in oligodendroglial cell lines that have lower *ABCD1* expression [260]. VLCFA accumulation might also cause demyelination by limiting peroxisomal synthesis of plasmalogens [261,262], notably plasmenyl-ethanolamine [261]. Plasmalogens are essential for OLs to initiate the membrane wrapping process [263].

A prominent role of oligodendroglial peroxisomes in the induction of brain pathology was demonstrated in a mouse model in which peroxisome biogenesis was impaired by the selective knockout of peroxin-5 (Pex5) in OLs [166]. This CNP-Pex5 mutant mouse provided a striking phenocopy of human cALD, with disease onset following normal developmental myelination. The brains of these mice showed strong VLCFA accumulation in myelin, severe axonal loss, severely lower plasmalogen levels, and early microglial increase in white matter, followed by demyelination with characteristic regional variability and inflammation with infiltrating perivascular macrophages and B and T cells. Information about the axons of the spinal cord tracts in CNP Pex5-KO mice is limited [166,264,265]. Another neuronal helix–loop–helix protein (NEX) promoter-driven Pex5 KO showed that the lack of peroxisomes in neurons did not lead to VLCFA accumulation or brain dysfunction, while another glial fibrillary acidic protein (GFAP) promoter-driven Pex5 KO showed that the lack of peroxisomes in astrocytes, despite causing VLCFA accumulation, had no significant impact on CNS functioning [264,266]. A mouse with a specific peroxisome KO in microglia is not available. Brain OLs are thus the key and only players responsible for the cALD-like pathology of CNP-Pex5 mutant mice. These findings highlight the indispensable role of peroxisome OLs in myelin maintenance. Hence, VLCFA accumulation appears to be a necessary but not sufficient factor for generating CNS pathology, at least in mice. The authors convincingly postulated that, while it takes several years for cALD patients’ OL peroxisomes to accumulate secondary functional impairments able to cause neurodegeneration [166], secondary peroxisomal changes might not have enough time to alter OL peroxisomes in the short-lived *Abcd1*^−/−^ mouse [152], which protected these mice against brain disease.

The effects of VLCFA accumulation have also been studied in other mouse models involving peroxisomal VLCFA degradation. Peroxisomal acyl-CoA oxidase (ACOX1) is the rate-limiting enzyme of VLCFA β-oxidation. *Acox1*-KO mice accumulate VLCFAs in their brains but have neither axonal loss [267] nor any neurodegenerative phenotype [268]. In contrast, children with *ACOX1* deficiency suffer devastating neonatal CNS disease, with VLCFA accumulation and early death [269], and *dACOX1* mutant *Drosophila* have glial and axonal loss [270]. The lack of disease in *Acox1^−/−^* mice is likely due to redundancy with murine *Acox2* and *Acox3*.

Hydroxysteroid-17-β-dehydrogenase-4 (HSD17B4) is another peroxisomal enzyme (“bifunctional protein”) that catalyzes two of the four reactions in FA β-oxidation. Ablation of the *Hsd17b4* gene caused severe brain pathology in mice [271]. These mice displayed severe coordination problems and massive neuroinflammation, which was less severe but still present in mice lacking *hsd17b4* specifically in neural cells (Nestin-Hsd17b4 mice) [272]. Together, these findings may suggest that peroxisomal β-oxidation is the major function of peroxisomes in the nervous system. Alternatively, impairment of peroxisomal β-oxidation may induce a secondary defect in peroxisomes, i.e., an additional loss of peroxisomal functions [253].

These findings indicate that various impairments in peroxisomal VLCFA β-oxidation induce brain pathology in humans, and that animal-model results must be interpreted with caution.

Drosophila with knockouts of enzymes involved in VLCFA production and degradation recently provided a new link between VLCFA accumulation and neuron degeneration or neuroinflammation [273] by showing the deleterious effect of S1P, a bioactive lipid generated by VLCFA accumulation in sphingolipids and irreversibly degraded by sphingosine-1-phosphate lyase (SGPL1) [274]. In vertebrates, VLCFAs are converted to C24 sphingolipids by ceramide synthase-2 (CerS2) [207], which is predominantly expressed in OLs [275]. C24-sphingomyelin accounts for 25% of brain sphingomyelin [216]. In *Drosophila*, S1P is produced by the glial cells that ensheathe axons, the equivalent of OLs in vertebrates. In vertebrates, OLs and microglia produce S1P [276,277], not neurons or astrocytes. In the fly brain, increased S1P—not VLCFA accumulation per se—triggers direct axonal loss, stimulation of immune cells, and neuroinflammation [273], meaning that VLCFA accumulation in OLs is noxious to the CNS, as they can transport S1P to axons [273]. Elevated S1P is also toxic in vertebrates [273]. Thus, S1P, which is increasingly produced by VLCFA-accumulation in OLs, appears as an ideal culprit for poisoning axons. The prominent OL S1P production could contribute to the difference between CNP-Pex5^−/−^ mice with severe cALD-like syndrome [166] and NEX- or GFAP-Pex5^−/−^ mice that have no detectable CNS pathology [264].

Another role of OLs is to provide axons with energy substrates. Because axons are not self-sufficient for their energy demand, OLs provide them with glucose and FAs for mitochondrial oxidation, with FAs being their major energy source [278].

OLs funnel energy substrates directly to the axonal space through myelinic channels [279]. Glucose, glycolytic substrates (lactate, pyruvate) [280,281], and ketone bodies (acetoacetate and β-hydroxybutyrate) [282,283] are transported from this space into axons by their glucose transporter protein type-1 (GLUT1) and monocarboxylate transporter-1 (MCT1) transporters, respectively [237]. When glucose availability is scarce, myelin synthesis declines and FA-derived acetyl-CoA supports mitochondrial respiration and OL survival [278]. This shift of normal myelin turnover to lipid-based ATP generation allows OLs to share more glucose-derived pyruvate/lactate with the axons to support ATP generation and prevent axon degeneration [278,280]. To perform all of their tasks, OL energy demand is enormous, reaching a maximum during early myelination and remaining at a high level throughout life [284]. The signals associated with neuronal activity that are sent to OPCs and OLs seem directly related to brain-area function and differ across brain cortex, brainstem, and spinal cord [285]. Glucose utilization and glycolysis are adapted to the energy demands of spiking neurons [286] that release glutamate signaling NMDA-expressing OLs to modulate OL generation, number, neuronal contacts, and myelin synthesis [287]. This adaptive metabolic homeostasis renders unlikely the hypothesis of mitochondrial energy deficiency as a primary mechanism of axonal degeneration in cALD or AMN [179,181,182,288]. However, it is possible that diminished axonal activity in certain brain regions, triggered for example by toxic lipid transfer from OLs, contributes to decreased signaling to OL, entering an axon-to-neuron vicious circle.

A prominent OL role in AMN pathogenesis is supported by the results of a gene-therapy study that used an adeno-associated vector (AAV) carrying the human *ABCD1* gene driven by the myelin-associated glycoprotein (MAG) promoter [289]. Soon after intravenous vector injection into *Abcd1*^−/−^ mice at day 10 of life, *ABCD1* peroxisomal expression was observed in ~50% of OLs and ~30% of astrocytes in the spinal cord white matter [172]. Two years later, *ABCD1* expression persisted in 6–7% of the OLs and 16–19% of the astrocytes, as would be expected of the prolonged expression of an episomal transgene in slowly dividing cells. VLCFAs decreased in the spinal cords of treated mice that had near-normal motor performances [172]. In a more recent study, the same vector was injected intra-cisterna magna at 18 months of age, when *Abcd1*^−/−^ mice start losing balance and motricity [176]. As early as 1–3 months post-injection, the treated mice had restored and near-normal motor performances, whereas untreated mice experienced neurological deterioration. At 24 months of age, hABCD1 expression was present in 22% in the white matter cervical spinal cord OLs and 22% of astrocytes. Punctate images of vector product showed its incorporation into peroxisomes [176]. No *ABCD1* expression was detected in motor cortex or DRG neurons, parental somata of the corticospinal and spinocerebellar tracts, or in the spinal cord or brain microglia [176]. Based on the surprisingly rapid vector effect, we postulated that the axonopathy of these mice was largely functional [176]; indeed, allowing *ABCD1* expression to be reinstalled in OL peroxisomes is likely to restore the function of crucial metabolic pathways [149]. The co-transduction of astrocytes may have played an additional role, as suggested by previous gene therapy attempts that used a vector targeting neurons and astrocytes, but this had a more limited effect than the OL-targeting vector [79,290,291].

### 6.2. Neurons and Axons

Axons’ enormous surface area makes them highly energy demanding to maintain their membrane potential. Therefore, neurons and axons face not only a huge energy demand [284] but also a problem of energy distribution throughout the tens of thousands kilometers of total myelinated axon lengths, given that the average axon is 20,000-fold longer than its parental cell body. For example, the corticospinal (pyramidal) tract, which is severely affected by AMN in humans and *Abcd1*^−/−^ mice, conveys energy-demanding motor messages from motor cortex neurons to the motor neurons of the lumbar spinal cord over a distance of dozens of centimeters in humans, <5 cm in mice. The longer axons, those in the spinal cord tracts, are the most energetically vulnerable. To meet energy needs, axons cannot import substrates directly, like most cells, because of the myelin barrier, except at Ranvier nodes, where they can take up glucose, lactate, and ketones via their GLUT1 and MCT1 [284]. They rely on astrocytes for the lactate supply and on OLs to provide glucose, lactate, and FAs through their myelinic channels to axons (see above).

While mitochondria fill dendrites and axons, peroxisomes are few in neuron perikarya or dendrites, rare within axons [251,253,254,255,292,293], and absent in synaptic terminals in the spinal cord [255] except during early development [255]. Because neurons have few peroxisomes, they do not play a major role in VLCFA degradation, as demonstrated by NEX-Pex5 mice that have no peroxisomes in their neurons and no VLCFA accumulation [264].

In many neurodegenerative diseases, including AMN [49,89,181,187,288,294,295,296,297], axonal pathology precedes cell body loss in the form of “dying back axonopathy”, in which axons gradually degenerate toward the cell body [298,299]. The prime suspect is mitochondrial damage and dysfunction and the ensuing cellular energy crisis [300]. Distal axon survival is dependent on axonal transport that is another energy-demanding process. The principal mechanisms of axon degeneration are metabolic stress, disturbed axonal transport, and mitochondrial dysfunction [301]. Failure of OLs’ energy-trophic function may contribute to such axonal neurodegeneration [302,303]. The dysregulation of axonal energy metabolism appears to be a major risk factor for axonal degeneration in AMN, like in other neurodegenerative disorders [304,305]. However, the cause(s) of such neuron and/or axon degeneration in X-ALD remain unknown.

Neuron-targeting gene therapy using the AAV9 capsid and the ubiquitous CBA promoter has provided valuable information about the potential role of several neuron populations in AMN pathology. Shortly after intrathecal injection of an AAV9–CBA–*hABCD1* vector into 3–5-month *Abcd1*^−/−^ mice, ALDP expression was found in spinal cord and DRG neurons, but not in OLs [290,291]. VLCFA accumulation was decreased by 9–24% in the spinal cord white matter [79,290,306]. Intracerebroventricular injection of the same vector induced *hABCD1* expression in spinal cord neurons, astrocytes, and microglia, but not in OLs [79,291]. In summary, restoration of *ABCD1* expression of the neuron populations targeted in these studies had no significant or consistent effect on motor performance. This does not rule out, but nevertheless questions, a contribution of these neurons to *Abcd1*^−/−^ mouse pathology. In addition, the cited routes of administration did not allow the vector to transduce neurons of the motor cortex from which corticospinal axons originate. The interpretation of these results is also hampered by the short-term design of these studies, and by the fact that motor test performances were unexpectedly found comparable in 20-month-old untreated *Abcd1*^−/−^ and wild type mice [79].

### 6.3. Microglia, Macrophages, Immune Cells

These cells are the effectors of the inflammatory processes that occur in cALD. Hence, it is essential to elucidate why and how neuroinflammation is initiated and becomes pervasive in cALD [77].

#### 6.3.1. Microglia

Microglia comprise 0.5–16.6% of the total cell population in the adult brain and spinal cord, depending on the anatomical region, sex, and stage of development, among other variables [307,308]. Our understanding of microglia biology in mice and humans has changed drastically in the recent years [309,310,311,312]. Self-renewal via proliferation and apoptosis maintains mature microglia through adulthood. Microglia live 0.5–3.4 years in the mouse brain [313] and up to 20 years in the human brain [314]. Their peroxisome content is abundant [166,253,315], and they express abundant ALDP in normal microglia in human and mouse brains [156]. Microglia are constantly surveilling what happens to maintain CNS homeostasis [316,317,318]. Microglia also support myelin formation and myelin integrity during CNS development and throughout life [319]. To do so, microglial processes constantly monitor myelin sheaths at Ranvier nodes [320], and myelin fragments are found within paranodal microglia [321,322]. Under physiological conditions, microglial numbers and spatial distributions are tightly regulated, resulting in a tile-like network that covers the entire neuropil [323]. Normal microglia can suppress neuroinflammation and protect brain tissue by releasing anti-inflammatory mediators, such as IL-10 and TGFβ (reviewed in [309]). That normal protective role is disrupted in response to CNS damage, inducing microglia to proliferate as reactive “microgliosis” [309]. Fine, markedly dynamic processes enable microglia to scan their environment, meticulously sensing signals to acquire specific functions [309,324]. Microglia constantly eliminate cell debris and dead cells through highly efficient phagocytosis using specific recognition, engulfment, and degradation processes to maintain brain homeostasis in health and disease [310,325,326,327]. ATP released by dying cells, a key signal, triggers microglial chemotaxis and engulfment. If this occurs during aging or disease states, axonal degeneration boosts microglial phagocytosis [328]. Microglia interact with OLs, astrocytes, neurons, and other CNS cells [312]. Age decreases microglia-mediated clearance of myelin fragments [329], but white matter-restricted microglia (WAM) are an age-dependent state able to clear age-associated damaged myelin [330]. Aged microglia also become more responsive to pro-inflammatory stimuli (“microglial priming”) (reviewed in [331]). The physiological, transcriptional, and phenotypic signatures of microglia are much more complex than the previously named M1 “resting” and M2 “activated” states, a simplified description still used by many [332]. Indeed, the microglia population is highly heterogeneous, comprised of cells in discrete transcriptional dynamic “substates” that appear in defined contexts and brain regions [333,334,335,336]. Microglia are different in the brain and spinal cord [334]. They are extremely versatile and respond immediately to subtle changes in CNS homeostasis and highly diverse stimuli. Due to this ability, they are involved in most CNS developmental, degenerative, and inflammatory processes and diseases [337,338]. Microglia can change their lipid metabolism in response to the lipid flux that occurs when neurons and OL die in neurodegenerative contexts [339].

While many microglial functions are protective in the healthy brain, some of these functions can become dysregulated and deleterious in disease contexts [312,326]. Thus, microglia are a double-edged sword. Microglia are highly versatile functionally and morphologically, and they can rapidly adapt in response to a diverse range of stimuli [340]. Upon detection of either pathogen-associated molecular patterns (PAMPs), damage-associated molecular patterns (DAMPs), or neurodegeneration-associated molecular patterns (NAMPs), microglia undergo rapid phenotypic change [341,342]. Under certain disease conditions, microglia can also be seen to proliferate and undergo morphological changes. That response, termed microgliosis, likely increases the ability of these cells to survey the brain parenchyma and to migrate more easily towards insults [340]. Indeed, under pathological conditions, microglia can acquire a detrimental pro-inflammatory phenotype that actively contributes to neuroinflammation, express genes involved with phagocytosis and lipid metabolism [343], and phagocytose unwanted material and synapses [344]. Activated microglia migrate to the site of damage via a process called chemotaxis and release chemokines (e.g., CCL2 and CXCL1), pro- and anti-inflammatory cytokines (e.g., IL-12, IL-10, TNF, TGF-β), and a variety of other inflammatory mediators [341]. These mediators further stimulate immune responses in other glia cells [345]. Microglia also present antigens to immune cells, inducing CNS inflammation [346]. They can also induce highly reactive A1 astrocytes that secrete a soluble toxin that directly kills neurons and OLs [347]. Activated microglia can drive demyelination [348]. During aging, axonal degeneration boosts microglial phagocytosis [328]. When it occurs, peripheral immune cell infiltration shapes microglia into a pro-inflammatory phenotype and accelerates disease progression.

Eichler et al. proposed that the microglial enrichment observed in the normal-appearing white matter of cALD patients could be due to the compensatory recruitment of these cells, while microglial apoptosis was observed in the non-lesioned zone of cALD (called “pre-lesional”) [178]. Because the microglia of *Abcd1*-KO mice, unlike normal microglia, cannot prevent the effects of low doses of C26:0-lysophosphatidylcholine on axonal degeneration, the authors hypothesized that VLCFAs might exert toxic effects on X-ALD patients’ microglia, which may hamper phagocytosis and other functions. When microglia fail to degrade VLCFA-enriched myelin fragments and undergo apoptosis, their contents may be released into surrounding tissue and trigger an inflammatory reaction [178]. Murine microglia mutated in the *Abcd1* and *Abcd2* genes contain lipid inclusions and accumulations of cholesterol and VLCFAs [349] similar to those observed in cALD patients’ brain macrophages. RNA sequencing revealed large-scale reprogramming of genes involved in lipid metabolism, immune response, cell signaling, and autophagy [350]. Following LPS exposure, the *ABCD1*-mutated microglia had numerous differentially expressed genes encoding pro-inflammatory cytokines or involved in phagocytosis, antigen presentation, and co-stimulation of T lymphocytes. Those changes were reflected by altered phagocytic capacity, increased redox-related inflammasome activation, increased release of inflammatory cytokines, including TNF, and up-regulated T-lymphocyte responses [351,352]. Due to their remarkable distribution in brain white matter and their strong regulatory role towards other cells, microglia have been proposed as the primary effectors of inflammatory processes that characterize cALD [178,353,354,355].

As for other CNS cells, cell and gene therapy introduced further insights into microglial involvement in X-ALD pathogenesis. The positive results of HSC transplantation into cALD patients [24,356] indicated a role for brain microglia because microglia were thought to originate in bone marrow at that time [357]. In fact, it is now known that the positive results of HSC transplantation were due to donor monocyte-derived macrophages that could migrate to the brain and limit inflammation when resident microglia are overwhelmed. Microglia were also the first target of gene therapy experiments in *Abcd1*^−/−^ mice [290,291,358]. Since microglia express ALDP [156,178], they were suspected of being involved in spinal cord pathology, despite the absence of inflammation [178,353,354]. If that were the case, importing *ABCD1*-expressing bone marrow HSCs would replace the failing mutant microglia. Early results using HSCs transduced ex vivo with lentiviral-*hABCD1* vector were said to be positive [358], but this remains unconfirmed. Moreover, the recent finding that cALD patients having undergone allogeneic HSC transplantation in childhood subsequently developed AMN [359] indicates that the AMN axonopathy pathogenesis is largely independent of phagocytic cells.

#### 6.3.2. Resident Macrophages

The yolk sac-derived precursors of resident macrophages are only in the perivascular compartment [360,361], where they maintain their cellular origin in adulthood [362,363,364]. The turnover half-time of resident macrophages averages 150 days [317]. They are located outside the parenchyma in the perivascular compartment surrounding arteries and veins, as they penetrate into the brain parenchyma (reviewed in [365]. Thus, perivascular macrophages function at the interface between blood and brain parenchyma as first responders in diverse neuroimmune interactions [366]. They belong to CNS border-associated macrophages (CAMs) [367,368]. Being directly exposed to peripheral stimuli, they can respond to inflammatory and chemical cues, including signals from microbiota [369] and mechanical cues [370]. As immune surveillants, CAMs clear harmful substances through phagocytosis, antigen presentation, and cytokine production [364,368,371,372,373,374,375,376], and they contribute to the maintenance of CNS homeostasis (reviewed in [377]). Single-cell profiling suggested functions shared with microglia and astrocytes in maintaining CNS homeostasis [372] and regulating barrier functions [367,378,379,380]. In addition, they control cerebral spinal fluid (CSF) dynamics and homeostasis [381].

CAMs may proliferate and undergo transcriptional and proteomic changes in response to various signals. With aging, or premature death, perivascular macrophages are replaced by monocyte-derived macrophages that appear to be more prone to induce inflammation [382,383,384]. Despite their critical role and the likely CAM involvement in human neurodegenerative diseases, data regarding their specific role remain scarce [377,384]. Although resident macrophages express ALDP abundantly [156], *ABCD1*-mutated CAMs could be suspected of suffering from its absence; however, to our knowledge, almost no specific data are available about resident macrophages in cALD.

Lipid-laden macrophages are a pathognomonic feature of peroxisomal disorders [385,386], likely due to the inability of these cells to fully degrade VLCFAs. Resident macrophage dysfunction, caused by lipid accumulation and impairment of peroxisomal function, may contribute to the pathogenesis of these diseases, including cALD. Compared to multiple sclerosis, only a few macrophages in cALD brain lesions express anti-inflammatory markers, whereas macrophages with pro-inflammatory markers are present in both diseases [355]. It was thus proposed that the inability of macrophages to convert into an anti-inflammatory repair state might underlie the inexorable demyelination in cALD, in contrast to the relapsing–remitting course of multiple sclerosis [355].

#### 6.3.3. Circulating Monocytes

Bone marrow-derived monocytes circulate in the blood, bone marrow, and spleen without proliferating. Skull marrow is uniquely connected to the CNS borders via skull channels, allowing for bidirectional exchanges of cells and signaling molecules between marrow and cells in the brain parenchyma [366,387]. Skull bone marrow cells continuously sample CSF to monitor brain status by accessing CSF-derived cues and thus can respond to CNS perturbations [388]. They can mount a tailored immune response [387,389]. They supply immune cells to the brain borders and parenchyma post-inflammation [388,389].

Circulating monocytes do not enter the healthy CNS in adulthood. In response to certain CNS diseases, they are recruited in affected areas. Understanding the cues that recruit immune cells from CNS-associated bone marrow will be critical for identifying mechanisms linked to this trafficking route and possibly involved in the neuroinflammatory mechanisms of neurodegenerative diseases [384]. These infiltrating monocytes expand locally and give rise to macrophages and inflammatory dendritic cells [390], but they are unable to generate resident microglia.

Upon CNS injury, monocytes respond to chemotactic factors by passing the blood–brain barrier (BBB) and migrating to sites of tissue damage. Once on-site, monocytes differentiate into macrophages and, together with CNS-resident microglia, reinforce defense mechanisms to clear the damaged site by producing neurotoxic cytokines and reactive oxygen species, and by phagocytosing myelin debris. Myelin uptake directly affects macrophage function by inducing an anti-inflammatory polarization state, which promotes tissue regeneration and remyelination [324]. Those two processes do not occur in cALD.

Monocytes normally strongly express ALDP and are most severely affected metabolically by defective β-oxidation and VLCFA accumulation [158]. CD34^+^ cells from cALD patients contain 4.7-fold more VLCFAs than normal [358]. In the profoundly inflammatory brain lesions, enlarged lipid-laden late macrophages indicative of prior myelin phagocytosis are prominent and are strongly positive for the pro-inflammatory marker CD86 [355]. A comparative analysis with acute multiple sclerosis showed a similar extent of pro-inflammatory activation but strikingly less anti-inflammatory CD206 and CD163 receptors expressed on cALD macrophages. Those authors’ whole-transcriptome analysis showed AMN monocytes’ normal macrophage differentiation and phagocytosis but a pro-inflammatory profile. Thus, *ABCD1* deficiency leads to impaired macrophage plasticity and incomplete establishment of anti-inflammatory responses. These findings emphasize that monocytes/macrophages are crucial therapeutic targets for preventing or stopping myelin destruction in cALD.

A recent study in rodent B lymphocytes showed that peroxisomal VLCFA transport is targeted by herpes viruses and antiviral host response [391]. *ABCD1* mutations might thus play a pivotal role of in viral infection and host defenses, prompting consideration of viral triggers in cALD and suggesting new candidate disease mechanisms [391]. It is also interesting that monocytes have been implicated as “Trojan horses” during viral infections, carrying infectious virus particles to immune-privileged sites and/or to sites protected by the blood–brain barrier (reviewed in [123]). It is not known whether ABCD1-mutated monocytes might carry viruses to the brain.

The implication of monocytes in cALD pathology is also highlighted by the results of cell and gene therapy in patients. The devastating inflammation and demyelination of cALD can only be stopped by the transplantation of a heterogeneous mixture of bone marrow progenitor cells and cells already engaged in differentiation. These HSCs can be extracted from a donor [24] or from the patient. If extracted from the patient, HSCs are transduced ex vivo by a lentiviral vector carrying the *ABCD1* gene [356]. Those implanted HSCs include a contingent of *ABCD1*-expressing circulating monocytes that then access neuroinflammatory areas of the brain through a leaky BBB. They are expected to home to the white matter, differentiate into macrophages, and phagocytize myelin debris, VLCFA-rich lipids, degenerated axon residues, and glial cell ghosts. In the short term, HSC transplantation reverses neuroinflammation and halts demyelination progression, as indicated by brain MRI [24,392,393]. It does not prevent the *ABCD1*-mutated OLs from continuing to produce toxic lipids that poison axons. We postulate that when the BBB recovers its function several months post-transplant and is no longer leaky, curative bloodborne circulating monocytes can no longer cross it. It is probable that the imported macrophage population, which has a limited lifespan, slowly declines and may no longer ensure efficient phagocytosis. OLs do not remyelinate the demyelinated brain areas. Hence, as would be expected, neuroinflammation recurs 5–10 years after HSC transplantation, as shown by the reappearance of gadolinium extravasation in demyelinated areas and demyelination progression [394].

Lauer et al. studied a brain autopsy specimen of a boy who succumbed to advanced cALD 15 months after undergoing allogeneic HSC transplantation [393]. Engrafted bone marrow-derived cells were present in the vascular and perivascular white and grey matter spaces. As expected, no detectable ALDP was present in astrocytes, OLs, or microglia [156], while striking characteristic punctate peroxisomal ALDP expression was found in circulating and perivascular monocytes, endothelium, and pericytes at the demyelinating lesion edge and surrounding cortex [393]. Those observations suggested that HSCs provided vascular elements to the cerebral microvasculature and restored the microvascular function in brain white matter. No information about this young patient’s spinal cord was available. The fact that HSC transplantation into cALD patients does not prevent AMN occurrence at an older age [359] indicates that HSCs either could not engraft in the spinal cord white matter or, more likely, that microglia pathology and HSC replacement are not involved in AMN pathogenesis.

### 6.4. Astrocytes

Interposed between neurons and the vasculature, each astrocyte is in contact with up to four capillaries and enwraps about four neuronal cell bodies and 10^5^ synapses [395,396,397]. Astrocytes are essential to neuron survival and function, and they are responsible for a number of functions relevant to CNS homeostasis. Indeed, they recycle neurotransmitters, e.g., removing excessive glutamate through their glutamate transporters. Astrocytes clear extracellular fluid potassium, contribute to synaptic plasticity and transmission, maintain the BBB, release lactate to fuel neurons and axons, buffer free radicals, and regulate blood flow (reviewed in [398,399]). Those reviews described astrocytes as highly plastic cells whose morphology and gene expression differ across the CNS. Subsets of reactive astrocytes can be developmentally induced or stimulus-induced [398,399,400,401,402]. The latter respond to neurotransmitters, cytokines, ions, physiological or pathological signals from microglia, neurons, OLs, other astrocytes, pericytes, and endothelial cells. The close connections formed between astrocytes with neurons enable sensitive detection of neuronal damage and effective regulation of the subsequent inflammatory response. Dying neurons release ATP and potassium, which can induce inflammasome activation within astrocytes [403]. Reactive astrocytes are a double-edged sword [404], as they can be homeostatic, adaptive, and beneficial or maladaptive and detrimental [405], failing to carry out their neuro-supportive roles [406] or driving pathological progression by releasing inflammatory cytokines. Microglia and astrocytes do not initiate inflammatory responses independently [407]. Instead, upon damage detection, microglia secretion of IL-1α, TNF, and complement component 1q (C1q) stimulates astrocytes to acquire a more reactive, inflammatory phenotype [347]. In turn, reactive astrocytes probably secrete additional factors that affect microglia-mediated neuroinflammatory behaviors [408]. Depending on the stimuli, distinct activation modes in these immune cells are initiated [409]. In response to multiple environmental stimuli, including those produced by microglia, astrocytes release a broad range of pro-inflammatory molecules, including chemokines (e.g., CCL2, CXC3L1, CXCL1) and cytokines (e.g., interferon-gamma (IFN-γ), IL-12, TNF, IL-10, and TGF-β). Those molecules open the BBB [410], attract inflammatory cells [347,411], and support immune cell invasion into the CNS or entry of molecules that exert suppressive effects [411]. Astrocytes can thus exacerbate or attenuate inflammation [412,413]. Astrocyte subsets express S1P receptors involved in demyelination and axonal loss. Thus, depending on the context, astrocytes are inflammation regulators and targets [399,406].

Astrocytes use ELOVL1 to elongate FAs into VLCFAs [347]. Their VLCFA-degrading peroxisomes are localized in cellular processes including the end-feet [166,254,414]. Astrocytes transfer their VLCFAs to OLs or microglia [347]. Astrocytes in GFAP-Pex5 KO mice have strong VLCFA accumulation, albeit inferior to that seen in the OLs of CNP-Pex5 KO mice [166,264]. Reactive astrocytes can upregulate VLCFA-rich phosphatidylcholines, which drive the death of neurons and OLs, probably via lipoparticle secretion [415]. Lactosyl-ceramide is a sphingolipid that promotes pro-inflammatory programs in astrocytes and decreases lactate production. VLCFA accumulation in astrocytes may also cause a lipotoxic response through 5-lipoxygenase activation [261].

Whether or not the *ABCD1* mutation changes the repertoire of astrocyte responses to extrinsic stimuli is key to understanding X-ALD phenotypes. How do astrocytes carrying the same *ABCD1* mutation respond to various signals by exacerbating or attenuating neuroinflammation, leading to cALD or AMN, respectively?

Gene therapy again has brought additional information. The role of astrocytes in AMN pathology is supported by gene therapy experiments in young *Abcd1*^−/−^ adult mice. It was previously observed that an AAV9-CBA-h*ABCD1* vector targeting neurons and/or astrocytes had only a partial effect on disease phenotypes, notably the motor deficits [79,290,291].

### 6.5. Endothelial Cells

These cells, as BBB constituents, are extremely important in cALD pathology. They abundantly express ALDP [156,157,416]. The results of studying autopsy brain tissue from cALD patients suggested that the BBB is compromised [417] and can contribute to the invasion of circulating monocytes and lymphocytes into brain parenchyma. In vitro, *ABCD1* inactivation suppressed tight junction proteins in human brain microvascular endothelial cells [417]. Brain perfusion studies in symptomatic and asymptomatic cALD patients revealed that microvascular physiology abnormalities preceded MRI-detected white matter changes [418]. Dynamic susceptibility contrast MR imaging showed that, in the area beyond the contrast enhancement, perfusion was decreased in all patients for whom follow-up MRI demonstrated cALD progression. This hypoperfused region matched the T2-hyperintense zone lying between contrast-enhanced and normally enhanced white matter, suggesting an abnormal distribution of extracellular fluid. When cerebral disease progresses, the contrast-enhancement region moves into areas of previously decreased perfusion, suggesting the shunting of perfusion from adjacent areas to sites of active inflammation, early tissue injury, or dysfunction of the neurovascular unit [419]. HSC transplantation normalizes white matter permeability and microvascular flow [393].

### 6.6. Schwann Cells

The selective loss of functional peroxisomes in Schwann cells of CNP-Pex5 KO mice does not induce structural myelin deficits [252]. However, the conduction velocity in isolated sciatic nerves of these mice is diminished and compound action potentials are lower, despite unchanged axon numbers and sizes [252]. The authors postulated that the inability to degrade VLCFAs in peroxisomes may cause lysosomal dysfunction in Schwann cells, accumulation of VLCFA-rich gangliosides in the juxta-paranodal membrane, and delocalization of membrane proteins in the apposed axons, thereby perturbing current propagation. They also highlighted a role for 2′,3′-cyclic nucleotide 3′-phosphodiesterase-multifunctional protein 2 [252]. These findings demonstrate that axons require intact peroxisomal lipid metabolism in the connected Schwann cells. However, unlike other authors [29], we did not find nerve conduction defects in our *Abcd1*^−/−^ mice [175].

### 6.7. Adrenal Cells

Primary adrenal insufficiency is common in patients with X-ALD or other peroxisomal disorders [420,421]. The mechanism whereby increased VLCFA levels lead to toxicity in the adrenal cortex is not well understood [422]. Cholesterol, along with saturated VLCFAs, accumulates in the fasciculata and reticularis zonae, which are responsible for the production of cortisol and androgens, respectively [423]. These accumulations start during fetal development [424]. Over time, this chronic accumulation is thought to trigger apoptosis and adrenal cortex shrinkage, leading to markedly decreased cortisol production [423]. The membrane of adrenal cells is disrupted by altered microviscosity when exposed to C26:0, which supposedly leads to impaired response to adrenocorticotropic hormone (ACTH) [425]. Alternatively, cortisol synthesis could be impaired by a relative lack of cholesterol necessary for its production [30,426]. Excess VLCFAs are stored within intracellular lipid droplets (LDs), which are dynamically synthesized or broken down in response to environmental signals or cellular needs. In adrenocortical cells, LDs are small, numerous, and consist of cholesterol esters used for steroidogenesis [427]. LDs have other important functions, including regulation of lipophagy, buffering excesses of potentially toxic lipids and misfolded proteins, and prevention of oxidative and endoplasmic reticulum stress [428]. To perform their functions efficiently, LDs communicate closely through contact sites with other cellular organelles, including peroxisomes [429,430]. Since the tethering of LDs to peroxisomes involves ABCD1 [431], it is tempting to postulate that LD–peroxisome communication is abnormal in X-ALD adrenals.

HSC transplantation to cALD patients does not restore adrenal steroidogenesis; thus, grafted patients with pre-existing adrenal insufficiency must continue cortisol supplementation post-transplantation. However, AAV gene therapy has the potential to bring therapeutic transgenes into the adrenal cortex using appropriate capsids and promoters [432], which opens the door to a yet unattempted restoration of *ABCD1* function in affected adrenals.

## 7. Lessons from X-ALD Patients’ Fibroblasts

Pathways and effectors of VLCFA metabolism have been extensively studied in cultured fibroblasts from X-ALD patients [31,136,138,146,208,433,434,435,436,437,438,439,440,441,442,443,444,445]. Although useful for the study of biochemical defects, these cells are not representative of neurons or glial cells and therefore could only provide limited biochemical insights into pathogenesis.

## 8. Previous Views of X-ALD Pathogenesis

Various hypotheses have been proposed over the past 50 years by X-ALD experts to explain the intricacy and time course of VLCA accumulation, myelin breakdown, axon degeneration, and neuroinflammation that underlie cALD pathogenesis. Early hypotheses focused on “a primary breakdown of myelin due to its instability by an excess of VLCFAs [17,82], followed by an inflammatory reaction that destroys the myelin” [446]. When defective peroxisomal VLCFA β-oxidation was discovered, peroxisomes entered the pathophysiological scheme [447]. The 1993 identification of the gene causing X-ALD did not change earlier views [23]. In 2000, Aubourg and Dubois-Dalcq focused on OL death, postulating that it was caused by VLCFA accumulation or inflammatory cells [448,449]. In 2010, VLCFA-rich gangliosides [450,451,452] and phosphatidylcholine [163,451,452] were suspected to be primary triggers of cALD. The same year, Singh and Pujol summarized the ongoing hypotheses in a “three hits” descriptive sequence of deleterious events [136]. cALD would be initiated by VLCFA excess and reduced plasmalogens leading to oxidative stress (first hit), which would generate inflammatory disease (second hit) with the participation of environmental, stochastic, genetic, and/or epigenetic factors. Subsequently, the cytokine and chemokine mediators of the inflammatory response further would cause generalized peroxisome loss or dysfunction, resulting in cell loss and progressive inflammatory demyelination (third hit) [136,165]. According to other investigators [31,260], “the primary consequence of VLCFA accumulation is disruption of cell membranes and contribution to the impairment of astrocytes and microglia. VLCFA-induced oxidative stress causes damage to proteins, microglial activation and apoptosis. In addition, VLCFA accumulation impairs the capacity of OL to sustain axonal integrity, resulting in axonal damage”. Thus “cALD cannot be explained by mutations in *ABCD1* alone… ABCD1 remains a susceptibility gene, necessary but not sufficient for inflammatory demyelination to occur… the molecular mechanisms responsible for full blown inflammation are only poorly understood” [288]. In 2015, decreased blood flow, BBB leakage, and endothelial cells entered the pathogenic dance orchestrating cALD [417,419]. More recently, epigenetic factors were suspected “to alter the transcriptional program driving an impaired OL differentiation and aberrant immune activation in X-ALD patients” [453]. In 2024, Yska et al. concluded that “few explanatory grand unifying theories had been proposed” [295].

Surprisingly, AMN, the main X-ALD manifestation, has received much less attention over the years than cALD.

## 9. Revisiting X-ALD Pathophysiology

X-ALD pathogenesis seems to be governed by two major mechanisms acting primarily in OLs: VLCFA accumulation, which causes AMN and is cALD permissive, and a secondary peroxisomal pathology, suggested to be a necessary added mechanism of cALD [166]. We have previously seen that a variety of *ABCD1*-mutated glial cells—microglia, macrophages, astrocytes—have the capacity to control or trigger the inflammatory processes leading to the AMN or cALD phenotypes, respectively.

Given that studies on VLCFA metabolism and peroxisomes have been carried out in humans, mice, or *Drosophila*, there are several major caveats to a unifying theory. First, the gene background, gene expression, enzymatic equipment, cell subpopulations, and neuronal activity in specific brain regions differ in the three species. Second, species lifespan has a major impact on pathogenesis. Indeed, seen from a cell or a peroxisome, the consequences of being exposed to accumulated VLCFAs or defective β-oxidation are likely to be different if this exposure lasts for few weeks, months, or many years. Also, time to symptom appearance is dictated by the aging of neural functions and potential environmental encounters.

### 9.1. Adrenomyeloneuropathy

#### 9.1.1. The Human Disease

**Figure 2:**
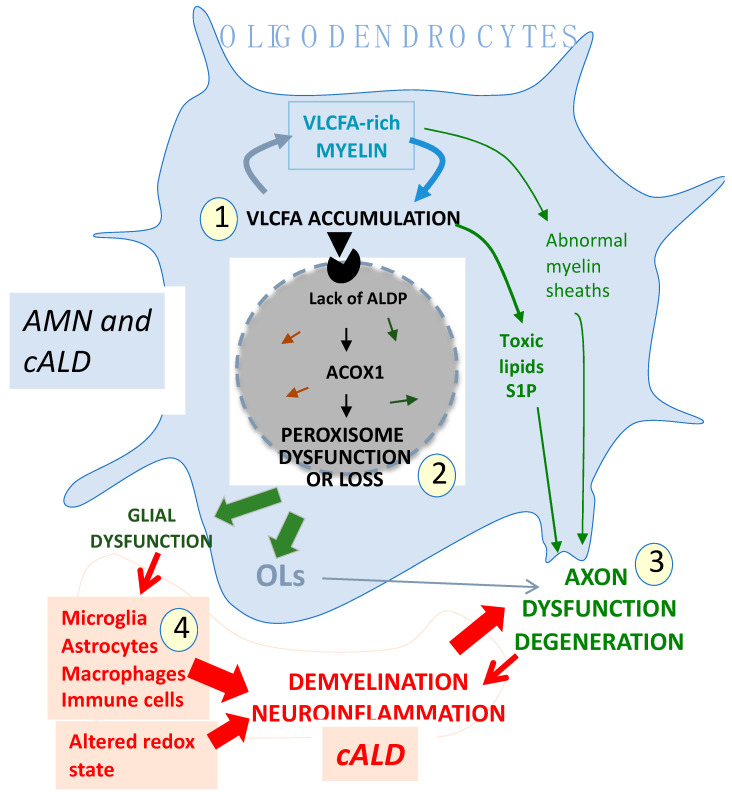
Simplified scheme of the main mechanisms that appear to contribute to X-ALD phenotypes. (1) The primary event is VLCFA accumulation in OLs and other glial cells that leads to synthesis of VLCFA-rich myelin and toxic lipids, e.g., sphingosine-1-P (S1P). (2) Peroxisomal dysfunction affects OLs and other glial cells. (3) Axon dysfunction and variable degrees of axonal degeneration. (4) In cALD patients, demyelination, brain inflammation, and axon degeneration are triggered by the subacute activation of microglia, astrocytes, macrophages, then extensive infiltration of the brain white matter by immune cells.

Myelopathy occurs in most, if not all, patients lacking *ABCD1* expression. We see it as the unavoidable adult phenotype of X-ALD, characterized by a spinal cord pathology progressing from childhood to adulthood and only interrupted by death in cALD patients. AMN starts with VLCFA accumulation in all cells that normally oxidize VLCFAs in their peroxisomes (Figure 2). OLs, by far the main VLCFA producers, accumulate most of them, because they are unable to degrade them and use them to synthesize toxic lipids, such as S1P. Given their close interaction with myelinated axons, OLs instill these toxic lipids into axons through myelinic channels. Other VLCFA-accumulation consequences in OLs include synthesis of myelinic VLCFA-rich gangliosides [166]. The abnormal myelin composition may progressively impair signal propagation in axons and decrease the supply of fuels, thereby impairing axonal oxidative metabolism and transport. Axon dysfunction slowly worsens in the CNS, reaching its maximum in the long axons of the spinal cord tracts, which leads to progressive manifestations in patients’ legs. In brain white matter, short axons are also likely to dysfunction, although to a much lesser degree. Is that milder dysfunction reflected by the mild decline of brain functions that occurs in AMN patients who still have normal brain MRI results? In the near future, it will be important to study patients screened positive at birth with thorough neuropsychological tests.

It takes decades for the axons of the column tracts of the spinal cord to degenerate and become atrophic in AMN end stages. Because the spinal cord was studied only at autopsy, pathogenic mechanisms have escaped investigation. Microglia may play a role in light of their pattern of increased activation and phagocytosis markers without pro-inflammatory signs in postmortem spinal cords of AMN patients [178]. Indeed, dysmyelinating foci are seen in AMN patients’ spinal cords and brains. Several factors can explain the absence of critical neuroinflammation in the CNS: limited axon degeneration, no severe peroxisomal dysfunction in OLs and other glial cells, only mildly perturbed redox state, and preserved homeostatic neuroprotective functions of microglia, resident macrophages, and astrocytes that may be resilient to inflammatory signals. These neuroprotective properties do not last forever in the fraction of AMN patients who develop inflammatory processes and demyelination later in evolution (“cerebral AMN”).

We have previously seen that the unique chimpanzee who developed typical cAMN died at 11 years, an age corresponding to 30–35 years in humans (the median life expectancy is estimated at 30.1 years for a captive male chimpanzee) [454].

#### 9.1.2. The Mouse Disease

In the *Abcd1*^−/−^ mouse, a short-lived species, spinal cord axons have a shorter length than humans and are exposed to deleterious mechanisms during less than 3 years. It is thus expected that 1.5 to 2 year-old-mice only show limited motor deficits. It would be important to follow the evolution of motor performances until death in this model and describe their final spinal cord pathology using classical and electron microscopy.

Inspired by the neuroprotective role of PPARγ agonists in several neurodegenerative diseases [455], investigators have tested the brain penetrant full PPARγ agonist leriglitazone in *Abcd1:Abcd2* double KO mice [456]. These mice have a severe form of axonal degeneration in the spinal cord and cerebellum and no cerebral pathology. At the highest dose, leriglitazone reduced axonal degeneration and microglia activation in the white matter of the spinal cord. Moreover, leriglitazone was able to improve the motor performance of the mice. Thus, leriglitazone appeared as a potential treatment for AMN. It was thus somewhat surprising that this drug has only been tested in patients with cALD (see next paragraph).

### 9.2. cALD

cALD occurs in a still unpredictable subset of *ABCD1*-mutated patients, most often children; rarely adolescents or young adults. We see its occurrence and subacute course as an “accident” of yet unknown causality in the otherwise progressive evolution of X-ALD towards AMN. The molecular events associated with the transition from VLCFA accumulation to devastating neuroinflammation are yet unclear. Due to *ABCD1* mutation, VLCFAs accumulate in OLs and other glial cells in the spinal cord and brain white matter (neurons do not oxidize VLCFAs). VLCFA accumulation in OLs generates toxic lipids that poison the many connected axons (Figure 2). The ensuing axonal dysfunction in the spinal cord will lead to AMN much later if HSC transplantation allows for survival of the young patients. While axon dysfunction progresses slowly in the spinal cord, things are quite different in the white matter of some brain regions where ALDP-expressing OLs are particularly abundant. Axon degeneration may be accelerated in these regions. The redox state may also be severely perturbed. Brain axon degeneration may induce clinical manifestations before MRI can be used to visualize demyelination. More or less independently from axonal degeneration and most often during childhood, neuroinflammatory processes typically start in certain brain regions. During a variable period, encompassing the pre-symptomatic phase of cALD, activated microglial phagocytosis succeeds in eliminating a slowly growing load of cell and myelin debris resulting from axon degeneration. This phase does not last forever. Soon, microglia, resident macrophages, and astrocytes become the actors of a stochastic “perfect storm”. The triggers of the inflammatory processes resulting from that storm are unknown.

In patients who will develop cALD, microglia switching to active states likely occurs in brain white matter, leading to reactive microgliosis in intact white matter areas of cALD patients. This microglial switch can be triggered at various ages, most often in childhood, by unknown factors that can be intrinsic or extrinsic to the brain, or systemic. Triggers could be viral infections able to activate microglia [457,458,459,460,461,462,463,464,465]. To our knowledge, no observations or investigations of potential links between viral infections and cALD have been reported. It is indeed remarkable that microglia disappear due to programmed cell death from perilesional white matter, where myelin and OLs are largely intact [354]. That microglia decay seems to precede the breakdown of myelin and loss of OLs [354].

In cALD children, demyelination typically starts in the genu of the corpus callosum, a region rich in ALDP-expressing OLs, then proceeds into the parieto-occipital lobes or other brain areas. Initial neurological deficits associated with non-inflammatory demyelinating lesions [288] are minor, but once the inflammation sets in, demyelination accelerates dramatically due to the ruptured BBB and infiltration of mononuclear, T-helper, and cytotoxic T cells and B cells. These often-intense lymphocytic infiltrates in the lesions, which are not typically seen in other metabolic leukodystrophies, are a very prominent and distinctive feature of cALD. Active demyelination and neuroinflammation spread into the white matter of typically affected brain regions, where VLCFA toxicity and degeneration of myelinated axons are the most advanced. The deleterious events can be followed with brain MRI. In core gliotic areas, microglia seem to mysteriously return to their normal distribution and re-express the homeostatic markers that were previously lost [353]. Secondary peroxisomal dysfunction may be a central mechanism of this neuroinflammation. Decreased plasmalogens may contribute. Possibly, the intrinsic accumulation of VLCFAs may weaken the proper neuroprotective capacities of microglia and astrocytes, generating pro-inflammatory signals.

Neither the mechanisms nor the primary causes of neuroinflammation are known. Among potential causes, variable expression of modifier gene products might explain cALD patients’ different evolutions towards brain neuroinflammation compared to AMN. It is also possible that undetected extrinsic events, like viral infections, trigger neuroinflammatory processes in predisposed children through their effect on certain brain microglial substates. Other environmental suspects are systemic inflammatory states or early adversity in its multiple facets, which might induce abnormal gene expression in microglia. Epialleles programmed in early embryonic or fetal life, or generated later, could also possibly orient microglia substates in the pathological direction and contribute to activating their participation in neuroinflammation and cytotoxicity (reviewed in [309]).

Given the expected role of PPARγ agonists to counter brain neuroinflammation [455], a small cohort study was performed in 13 adult patients with early cALD. The initial results suggested that leriglitazone could halt neuroinflammation and disease progression up to 2 years [466,467]. In a larger randomized trial, however, the decline in walking distance remained comparable in the leriglitazone and the placebo groups [468,469].

In summary, the predominant inflammatory component of cALD should not obscure the fact that primitive mechanisms are not restricted to cells responsible for neuroinflammation. This is clearly demonstrated by the dramatic recurrence of cALD within ten years following transplantation of lentivirus-transduced HSCs [394].

We believe that the other glial cells carrying the *ABCD1* mutation that have not been corrected are, in fact, very likely to be responsible for the relapse.

## 10. Future of Research on X-ALD Pathogenesis

Hopefully, technological advances will contribute to expanding our knowledge of pathological mechanisms. However, will we be able someday to understand the reasons for the fatal brain damage that affects a fraction of children harboring mutated *ABCD1*? And, could we explain the precise mechanisms of the disabling damage that develops more or less slowly in adulthood? With what research approaches? Classical analysis of post-mortem nervous tissue and studies in genetically modified animal models have been invaluable in understanding disease processes. However, *Abcd1*^−/−^ mouse models are unable to faithfully replicate human mechanisms and disease course, notably the neuroinflammatory responses [470]. The advent of human in vitro models of neural cells now provides unique insight into disease biology as a manipulable model system obtained directly from patients, as well as transcriptomics of cell populations and functional in vivo imaging.

### 10.1. Multi-Omics

In six *ABCD1*-mutated brother pairs discordant for the presence of cALD, the multi-omic profiling of blood samples including genome, epigenome, transcriptome, metabolome/lipidome, and proteome profiling was unable to identify statistically significant candidate molecular markers able to differentiate AMN and cALD patients [112].

### 10.2. Human Induced Pluripotent Stem Cells (iPSCs)

IPSCs are artificial stem cells formed from somatic cells (e.g., fibroblasts or blood) through the transient expression of reprogramming factors that stimulate de-differentiation to a pluripotent state, which is similar to that of embryonic stem cells. The modeling of monogenic diseases often relies on gene-edited human iPSCs compared with isogenic non-mutant controls. For modeling genetically complex disorders, which often involve multiple unknown loci, the use of patient-derived iPSCs is better adapted to study than genome editing. Once formed, iPSCs are capable of infinite self-renewal and can differentiate into specialized cell types. The derivation of iPSCs from multiple patients enables the analysis of similar mutations in diverse genetic backgrounds.

Patient-specific iPSCs are able to model genetic variability and susceptibility across patients, offering a personalized approach to understanding disease pathology. iPSC-derived cells provide a direct and consistent source of human OLs, microglia, astrocytes, and endothelial cells, enabling investigation of human-specific responses. iPSC-derived cells can be studied in monoculture (single-cell type), two-dimensional co-culture (multiple cell types), or three-dimensional (3D) culture (e.g., neural organoids) (reviewed in [403,471]). In addition, iPSC-derived cells can be studied after transplantation into the brain of an immunodeficient mouse (xenotransplantation) (reviewed in [403]). Phenotypes and mechanisms could first be investigated in iPSC monoculture models, then further validated in increasingly more complex cultures. While moving towards higher levels of iPSC model complexity is thought to improve the ‘brain-like’ authenticity of disease mechanisms, little available evidence currently supports this idea [403]. Indeed, the intricate network of specific interactions among the diverse cell types in a specific diseased or damaged brain or spinal cord white matter are not likely to be mimicked when iPSCs are studied in isolation and in the artificial conditions of cell culture [472].

Whereas remarkable progress has been made over the past decade, iPSC technology is still in its infancy, and notable challenges need to be addressed [473]. A critical evaluation of iPSC models reveals several drawbacks: primarily, these iPSC models often resemble a fetal state rather than an adult state, which is a key difference from the intended application of these models. Moreover, the reprogramming technique used for obtaining clonal iPSCs modifies the primary epigenetic marks present in patient’s fibroblasts [474]. Moreover, given that each cell category within the body and the brain possesses a distinct epigenome [475,476], it is implausible that a fibroblast epigenome can serve as a reliable surrogate for that of an OL, an astrocyte, or microglia, because the epigenomic marks that underlie the differences in gene expression across brain cells are not likely to be present in fibroblasts. In addition, many epigenomic marks may have been lost during reprogramming.

Below, we briefly summarize—cell type by cell type—the few results obtained from X-ALD patients. iPSCs derived from patients with cALD or pure AMN were used with the objective of unraveling significant molecular differences (biochemical phenotypes, levels of gene expression) that would help understand these two main disease forms.

At the pluripotent stem cell stage, early-passage iPSCs derived from AMN or cALD patients did not have elevated VLCFA levels compared to healthy controls in two studies [477,478], whereas elevated VLCFA levels in iPSCs from cALD patients were found in another study [479]. Undifferentiated iPSCs from two cALD patients showed dysregulated expression of genes involved in peroxisome abundance and neuroinflammation [478].

AMN patient-specific iPSC-derived OLs showed elevated VLCFA levels/, compared to healthy controls, but lower levels than those derived from cALD patients in two studies [477,478], as well as normal VLCFA levels in a third study [479].

Astrocyte and microglia iPSC models partially recapitulate the transcriptomes of their in vivo counterparts and may capture authentic neurodegenerative disease phenotypes [345,406,480,481,482,483,484]. cALD iPSC-derived astrocytes from two patients with cALD expressed higher levels of proinflammatory cytokines than those from two AMN patients and two healthy controls with LPS stimulation or without [477]. Those iPSC-derived astrocytes accumulated VLCFAs and showed different mitochondrial bioenergetics, cytokine-gene expression, and differences in signal transducer and activator of transcription-3 (STAT3)- and AMP-activated protein kinase (AMPK) signaling between two AMN and two cALD patients [485]. We know of no study of iPSC-derived microglia in X-ALD.

Functional and molecular studies on iPSC-derived resident macrophages are possible [486,487,488,489,490] but have not been studied in X-ALD patients.

iPSC-derived microvascular endothelial cells from cALD patients exhibited only half-normal transendothelial electrical resistance and LD accumulation [491].

Up to now, the few studies in X-ALD have relied on comparisons between only one or two patient-specific iPSC lines and a corresponding number of iPSC lines derived from healthy donors. When more iPSC lines become available, as for other neurodegenerative disease cell repositories [492], results will hopefully become more robust.

### 10.3. Single-Cell and Spatial Transcriptomics

These techniques have been applied to postmortem brains from many patients with neurodegenerative diseases [493,494]. Studies on microglial transcriptomic, proteomic, and epigenomic characteristics in specific contexts are beginning to reveal consistent, discrete responsivity patterns in health and disease. A trans-omic approach matching miRNA and metabolomics in the postmortem brains of five patients suggested the involvement of specific molecular and metabolic pathways in cALD [495].

Further information will likely come in the future from transcriptomes, miRNAs, and epigenomes of OLs, microglia, and other cells extracted from the apparently normal or affected areas of the brain and spinal cord white matter of cALD or AMN patients. Notably, one could obtain primary microglia from fresh postmortem brain tissues of patients with cALD or AMN, identify the different microglia clusters characterizing the disease, and study their functions. However, since microglia and astrocytes are highly responsive to their environment, removal of primary cells from the brain and further in vitro culture may skew these cells towards a more pro-inflammatory state that could affect subsequent experimental procedures.

### 10.4. Environmental Research in Mice

Traumatic brain injury may be a significant environmental risk factor for several progressive neurodegenerative disorders [496]. It has been implicated in a few anecdotical but striking observations of cALD occurrence following a head trauma [43,45,46]. Interestingly, the corpus callosum white matter has been shown to be the most sensitive region to repetitive head trauma [497]. Head trauma is known to cause microglial activation and neuroinflammation [498,499,500,501] and is amenable to experimental research in mice [502,503].

Another environmental approach would be to infect the brains of *Abcd1* KO mice with viruses [465,504,505] and to follow the effects of neuroinvasion on brain cell types, notably microglia and macrophages, on VLCFA accumulation and on motor phenotype.

### 10.5. Non-Human Primates (NHPs)

Modifications with genetic technologies would provide extremely valuable models to investigate neurodegenerative diseases [506,507], including X-ALD, given their close proximity to humans.

### 10.6. CNS Imaging

The most challenging aspect in the study of microglia and astrocytes is that the cells are not visible during life. We have seen that iPSC-derived cells are usually derived from non-cerebral cells and studied out of the tissue in which they act. Imaging techniques have been developed to non-invasively assess microglia and astrocyte activity in the human brain.

Positron emission tomography (PET) imaging involves the administration of a radioactive PET ligand, which will cross the BBB and recognize specific receptors in the brain. This technique can show neuroinflammatory parameters in the brain in vivo [508]. These radioactive ligands release positrons that annihilate into pairs of gamma rays detected by scintillators to show the 3D distribution of receptor-bound ligands. Indeed, these ligands bind to receptors whose expression is related to the activation of microglia (TSPO ligands) and astrocytes (PET tracers targeting monoamine oxidase B (MAO-B), which is upregulated in activated astrocytes and TSPO ligands). Other common targets developed for the detection of astrocytosis using PET are the type-2 imidazoline receptors (I2Rs).

MRI is another method in which neuroinflammation can be detected. Specialized MRI techniques can measure the diffusion of water within tissue and highlight areas of gliosis, where water diffusion is affected by alterations in cell morphology during neuroinflammation. MRI has been less utilized than PET to assess gliosis due to its lack of cell specificity. Interestingly, a study using diffusion-weighted MRI built a microstructural model of diffusion based on the ramified morphology of glial cells [509]. The model was tested on rats under several conditions and was validated for glial cells detection via immuno-labeling of the rat brain for microglia and astrocytes (GFAP). Interestingly, the model appears not only to be able to distinguish between microglia and astrocytes but also to assess the presence of neurodegeneration [510]. However, better cellular specificity might be achieved by measuring the diffusion of cell-specific endogenous metabolites with magnetic resonance spectroscopy [511,512,513].

## 11. Temporary Conclusions

The emergence of technologies able to enhance our understanding of X-ALD pathogenesis is promising, but there is still a long way to go. Obtaining information on the different cell types involved in primary disease mechanisms and their spatiotemporal implication is a nightmarish challenge. If one derives iPSCs from patients’ fibroblasts, he could obtain information on gene expression in an artificially differentiated cell type outside of its spatiotemporal context. If he studies CNS postmortem specimens, he can only analyze advanced stages of the two disease forms, be it cALD brain or AMN spinal cord. The limited incidence of X-ALD precludes hope of applying new cellular and molecular technologies to several dozen patients, as for Parkinson’s or Alzheimer’s disease, unless international repositories of iPSCs and postmortem samples are established and span many years. Systematic research of modifier gene variants using blind whole-genome research requires thousands of patients. Blind environmental studies that would prospectively investigate the first years of life of patients screened at birth will also require thousands of patients. Accidental environmental factors, such as head trauma or viral infections, may be key pathogenic mechanisms and should not be neglected by cALD researchers. Two potential avenues of progress could provide great advances, but they face enormous technical obstacles. The first one is the creation of NHP models in which *ABCD1* expression would be inactivated in one or another CNS cell type. The second is the functional imaging of patients studied from birth (neonatal diagnosis) to death, able to provide information about the microglial metabolism, neuroinflammation and activation, and astrocyte reactivity at different disease stages.

In conclusion, pessimism should not be an option for X-ALD researchers, even if they will have to continue searching for needles in a haystack without neglecting any avenue of clinical or experimental investigation. They will have to apply the famous maxim of Guillaume d’Orange (1533–1584), “il n’est pas nécessaire d’espérer pour entreprendre, ni de réussir pour persévérer” (You don’t have to hope to start, or succeed to keep going).

## Abbreviations

AAVs	adeno-associated vectors
ABC	ATP synthase (ATP)-binding cassette
*ABCD1*	ATP-binding cassette subfamily B member-1 gene
*ACOX1*	acyl-CoA (coenzyme A) oxidase gene
ACTH	adrenocorticotropic hormone
ALDP	ALD protein
AMN	adrenomyeloneuropathy
AAP	amyloid precursor protein
BBB	blood–brain barrier
cALD	cerebral ALD
C1q	complement component 1q
CAMs	CNS border-associated macrophages
CAT	catalase
CBA	chicken β-actin
CE	cholesterol esters
CerS2	ceramide synthase 2
CNP	2′,3′-cyclic nucleotide 3′-phosphodiesterase
CNS	central nervous system
COVID-19	coronavirus disease-2019
CSF	cerebral spinal fluid
CYP4F2	cytochrome P450 family 4 subfamily F member 2
*dACOX1*	*Drosophila* mutant acyl-CoA oxidase gene
DAMPs	damage-associated molecular patterns
Dbp	D site-binding protein
DRG	dorsal root ganglia
ELOVL1	very-long-chain-fatty acid elongase-1
ER	endoplasmic reticulum
FLAIR	fluid-attenuated inversion recovery MRI sequences
GFAP	glial fibrillary acidic protein
GLUT1	glucose transporter protein type-1
GPX1	glutathione peroxidase 1
HSC	hematopoietic stem cells
HSD17B4	hydroxysteroid-17-β-dehydrogenase-4
I2Rs	type-2 imidazoline receptors
IFN-γ	interferon-γ
IL	interleukin
iPSCs	induced pluripotent stem cells
KO	knockout
LD	lipid droplets
MAO-B	monoamine oxidase-B
MCT1	monocarboxylate transporter-1
MFGE8	milk fat globule-epidermal growth factor 8
MFP2	multifunctional protein-2
MRI	magnetic resonance imaging
NAMPs	neurodegeneration-associated molecular patterns
NEX	neuronal helix–loop–helix protein
OL	oligodendrocyte
OPC	oligodendrocyte progenitor cell
PAMPs	pathogen-associated molecular patterns
PET	positron emission tomography
Pex5	peroxin-5
Pex19p	peroxisomal biogenesis factor-19
*pmp-4*	peroxisomal membrane protein-4 gene
PMP70	70 kDa peroxisomal membrane protein
PNS	peripheral nervous system
*R/GCP*	red/green color vision gene
S1P	sphingosine-1-P
SOD2	superoxide Dismutase 2
TGF-β	transforming growth factor-β
TNF	tumor necrosis factor
TREM2	triggering receptor expressed on myeloid cells 2
TSPO	translocator protein
VLCFA	very-long-chain fatty acids
WAM	white matter-restricted microglia
X-ALD	X-linked adrenoleukodystrophy
